# Evaluation of the *Leishmania* Inositol Phosphorylceramide Synthase as a Drug Target Using a Chemical and Genetic Approach

**DOI:** 10.1021/acsinfecdis.4c00284

**Published:** 2024-07-18

**Authors:** Edubiel A. Alpizar-Sosa, Flavia M. Zimbres, Brian S. Mantilla, Emily A. Dickie, Wenbin Wei, Gabriela A. Burle-Caldas, Laura N. S. Filipe, Katrien Van Bocxlaer, Helen P. Price, Ana V. Ibarra-Meneses, Francis Beaudry, Christopher Fernandez-Prada, Philip D. Whitfield, Michael P. Barrett, Paul W. Denny

**Affiliations:** † Department of Biosciences, University of Durham, South Road, Durham, DH1 3LE, U.K.; ‡ School of Infection and Immunity, College of Medical, Veterinary and Life Sciences, University of Glasgow, Glasgow G12 8TA, U.K.; § Departamento de Bioquímica e Imunologia, Universidade Federal de Minas Gerais, Caixa Postal 486 31270-901, Belo Horizonte, Minas Gerais, Brazil; ∥ York Biomedical Research Institute, Hull York Medical School, University of York, York YO10 5NG, U.K.; ⊥ School of Life Sciences, Keele University, Staffordshire, ST5 5BG, U.K.; # Département de Pathologie et Microbiologie, Faculté de Médecine Vétérinaire, Université de Montréal, Saint-Hyacinthe, Quebec J2S 2M2, Canada; 7 Département de Biomédecine, Faculté de Médecine Vétérinaire, Université de Montréal, Saint-Hyacinthe, Quebec J2S 2M2, Canada

**Keywords:** *Leishmania*, inositol phosphorylceramide synthase, clemastine fumarate, polyomics, CRISPR-Cas9, thermal proteomic profiling

## Abstract

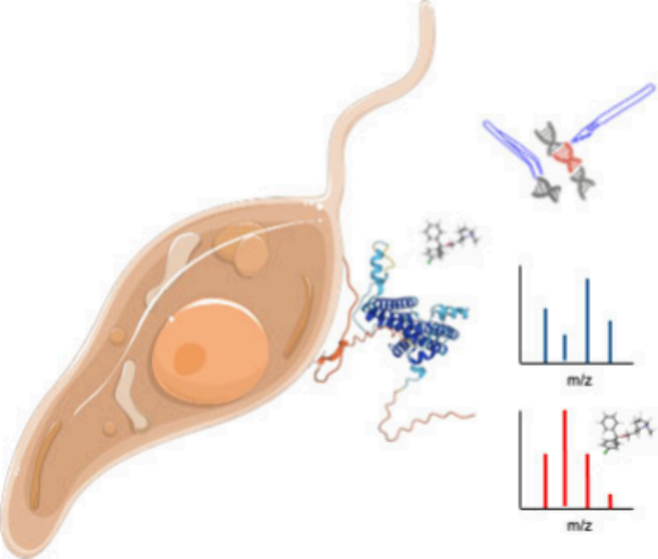

The lack of effective vaccines and the development of resistance to the current treatments highlight the urgent need for new anti-leishmanials. Sphingolipid metabolism has been proposed as a promising source of *Leishmania*-specific targets as these lipids are key structural components of the eukaryotic plasma membrane and are involved in distinct cellular events. Inositol phosphorylceramide (IPC) is the primary sphingolipid in the *Leishmania* species and is the product of a reaction mediated by IPC synthase (IPCS). The antihistamine clemastine fumarate has been identified as an inhibitor of IPCS in *L. major* and a potent anti-leishmanial *in vivo*. Here we sought to further examine the target of this compound in the more tractable species *L. mexicana*, using an approach combining genomic, proteomic, metabolomic and lipidomic technologies, with molecular and biochemical studies. While the data demonstrated that the response to clemastine fumarate was largely conserved, unexpected disturbances beyond sphingolipid metabolism were identified. Furthermore, while deletion of the gene encoding *Lmx*IPCS had little impact *in vitro*, it did influence clemastine fumarate efficacy and, importantly, *in vivo* pathogenicity. Together, these data demonstrate that clemastine does inhibit *Lmx*IPCS and cause associated metabolic disturbances, but its primary target may lie elsewhere.

Neglected tropical diseases (NTDs), a group of 25 diverse diseases,^1,2^ are classified as “neglected” due to their predominance in regions of poverty, as well as their relatively low priority on national and international health agendas.^3^ Causing a loss of an estimated 3.32 million disability adjusted life years (DALYs), accounting for 13% of all NTD DALYs,^4,5^ leishmaniasis is endemic in over 90 countries, impacting at least 12 million people per year, with over one billion people living at risk of the disease.^6^ The causative *Leishmania* species are sand fly borne kinetoplastid protozoan parasites,^7^ and infection via insect bites leads to a wide spectrum of disease, from self-healing but scarring cutaneous leishmaniasis (CL) to fatal visceral disease (VL). This disease diversity is dependent upon the infecting *Leishmania* species and host genetic background and immunity.^8^ While amphotericin B treatment initiatives in South Asia have significantly reduced global VL over the past decade, conflict-driven migration has sharply increased CL.^9^ Despite success, amphotericin B has severe side effects,^10^ and clinical resistance has been observed, at least in immunocompromised patients.^11^ Pentavalent antimonials (sodium stibogluconate [Pentostam] and meglumine antimoniate [Glucantime]),^12,13^ remain the frontline in CL treatment but also have major side effects,^14^ require parenteral^15^ administration and face rapidly emerging drug resistance.^16^ Reflecting on this situation, there is an urgent need for novel anti-leishmanial treatments that are inexpensive and free of side effects.

Recent work has identified the over-the-counter antihistamine clemastine fumarate as a potential anti-leishmanial drug candidate.^17^ This compound demonstrates polypharmacology in *Leishmania* spp; however, inositol phosphorylceramide synthase (IPCS) is clearly inhibited.^17,18^ This enzyme is not found in mammals which use sphingomyelin (SM) as their main sphingolipid, while fungi, plants, and some protozoa, including *Leishmania*, encode an IPCS to synthesize IPC.^19−23^ IPCS catalyzes the transfer of phosphoinositol from phosphatidylinositol (PI) to ceramide, whereas the mammalian equivalent facilitates the transfer of phosphocholine from phosphatidylcholine (PC) to generate SM.^18,24−30^ IPCS (AUR1p in fungi) has been shown to be a druggable target for antifungals,^24−27,30^ a potential herbicide target in plants^31,32^ and investigated as possible new antileishmanial target.^17,33,34^

In this report we re-evaluate the drug target status of the *Leishmania* IPCS, using a chemical approach with the previously identified inhibitor clemastine fumarate,^17^ and a genetic approach using the now well established CRISPR/Cas9 system in *L. mexicana*.^35^ The data demonstrate that clemastine fumarate is active against *L. mexicana*, as it is against *L. major*, *L. donovani*, *L. infantum* and *L. amazonensis*, and inhibits *Lmx*IPCS as well as *Lmj*IPCS as previously described.^17^ The *L. mexicana* metabolomic and lipidomic fingerprints following clemastine fumarate exposure were similar to those reported for *L. major*. Resistant lines generated and analyzed also demonstrated a similar pattern of mutations to those previously reported.^17^ However, utilizing a metabolomic and thermal proteomic approaches, the TCA cycle was identified as a possible target.

Furthermore, deletion of the encoding enzyme, *Lmx*IPCS (*LmxM*.34.4990) was achieved with viable insect stage promastigote parasites growing and transforming to mammalian stage amastigote forms at an equivalent rate to the parental line. While these cells demonstrated altered clemastine fumarate sensitivity, the data contradicted the status of IPCS as an antileishmanial drug target. However, gene deletion did diminish the abundance of the soluble inositol polyphosphates IP_2_ and IP_6_, indicating a role for IPC in the generation of these signaling molecules. Furthermore, *in vivo* analyses demonstrated a potential role for IPCS in *L. mexicana* pathogenicity, in contrast to *L. major*.^33^ However, while the complemented “add back” line largely recovered levels of IPC, pathology was not restored, correlating with an incomplete restoration of IPC levels and, unexpectedly, cardiolipins.

Overall, this work demonstrated that clemastine fumarate has a comparable mode-of-action in *L. mexicana* and *L. major*, although IPCS inhibition represents only one part of this polypharmacological effect.^17^ Furthermore, ablation of *Lmx*IPCS disables the parasite *in vivo* and thus reduces pathogenicity, supporting the status of this enzyme as a potential drug target.

## Results

### *Leishmania mexicana* Inositol Phosphorylceramide Synthase (*Lmx*IPCS) Is Functional and Inhibited by Clemastine Fumarate, a Slow Acting Anti-Leishmanial

**Figure 1:**
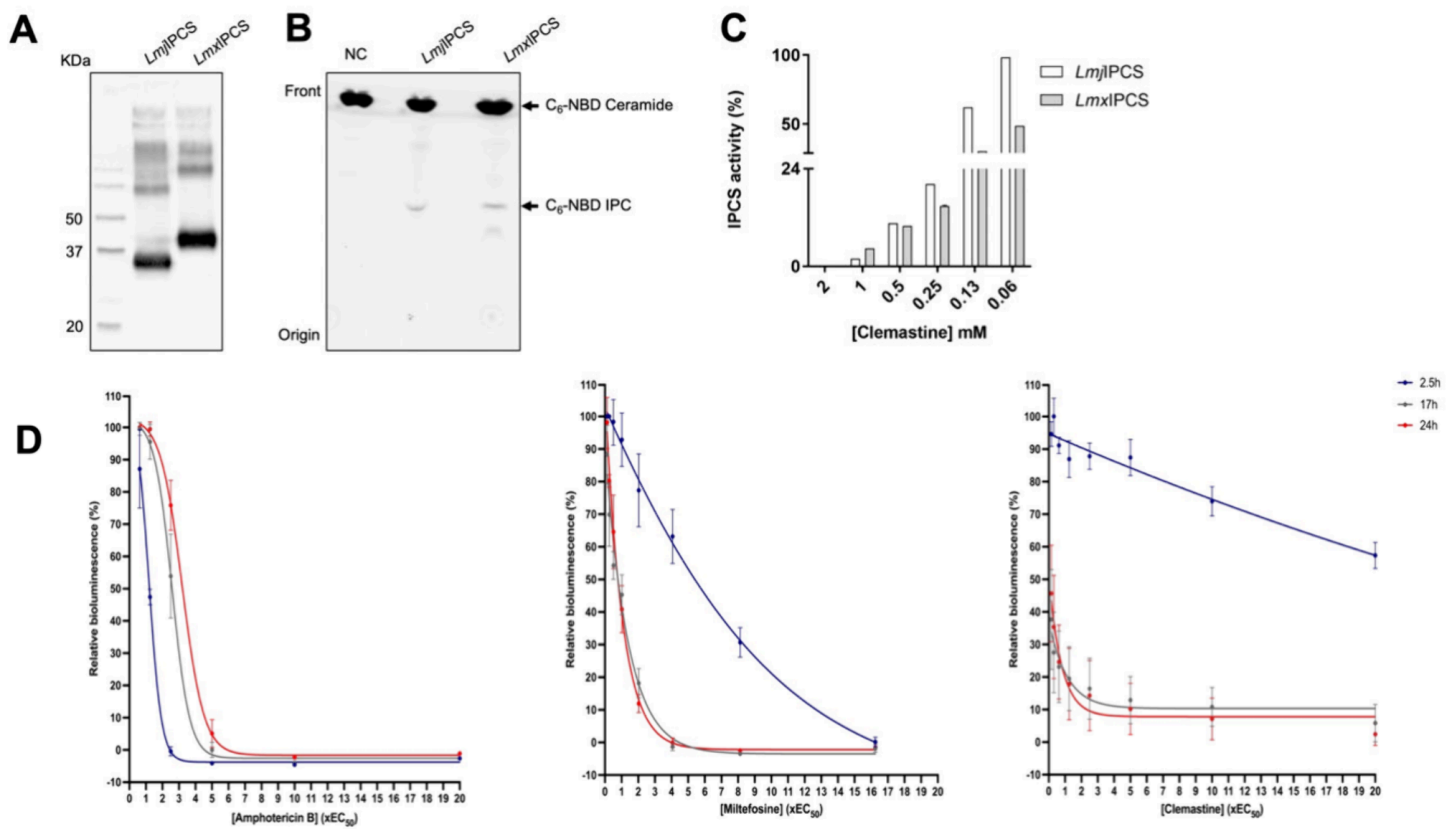
The *Leishmania mexicana* putative inositol phosphorylceramide synthase (IPCS) is functional and inhibited by clemastine fumarate. (A) The *in vitro* expression of IPCS from *L. major* (*Lmj*IPCS) and *L. mexicana* (*Lmx*IPCS) using wheat germ extract was confirmed by Western blotting; the expected sizes of *Lmj*IPCS and *Lmx*IPCS were 36 kDa and 43 kDa, respectively. (B) *In vitro* assay^17,34^ of these proteins expressed as proteoliposomes demonstrated their IPCS activity when visualized following thin layer chromatography (TLC). (C) The proteoliposomes with *Lmj*IPCS and *Lmx*IPCS were incubated with different concentrations of clemastine (mM) and the efficacy analyzed using the *in vitro* assay as in (B). The products of two independent experiments were quantified by ImageQuant software, and the graph was made in GraphPad Prism. (D) Rate of kill curves of amphotericin B, miltefosine, and clemastine fumarate against NanoLuc-PEST promastigotes.^17,34^ Bioluminescence readings taken at 2.5, 17, and 24 h normalized against internal control readings and plotted as mean ± 95% CI (amphotericin B, *n* = 3; miltefosine and clemastine, *n* = 6). Graphs were plotted in GraphPad Prism.

In previous studies we utilized a yeast-based complementation system to study the *L. major* orthologue (*Lmj*IPCS).^17,18,28,29^*Lmx*IPCS, however, failed to rescue a *Saccharomyces cerevisiae* AUR1 mutant (data not shown). Therefore, we validated *Lmx*IPCS as a functional orthologue of *Lmj*IPCS using a cell-free expression system to isolate these complex integral membrane proteins in functional forms within proteoliposomes (Figure 1A and B). This defined *in vitro* system allowed us to assay the efficacy of clemastine against *Lmx*IPCS and *Lmj*IPCS. Notably, *Lmx*IPCS (*LmxM.*34.4990) is larger than *Lmj*IPCS (*Lmj*F.35.4990) (Figure 1A) - 383 versus 338 amino acids due to a C-terminal extension of unknown function. Similarly, the orthologue in *L. donovani* (LdBPK.35.2.005030.1; 385 amino acids) has this extension, and we have previously noted that this also did not complement the yeast system employed for the study of *Lmj*IPCS^17,18,28,29^ (Norcliffe et al., unpublished). Dose–response assays revealed that *Lmx*IPCS was more sensitive to clemastine fumarate than *Lmj*IPCS (Figure 1C). However, with efficacy in the high micromolar range for both orthologues, compared to the low micromolar range for *Lmj*IPCS in yeast-derived micelles,^17^ it was clear that this lipid-rich *in vitro* platform masks the full inhibitory value of clemastine fumarate. To gain further understanding of clemastine fumarate as an anti-leishmanial candidate, taking advantage of an established NanoLuc-PEST system in *L. mexicana* as a dynamic indicator of cell viability,^36^ we examined the time-to-kill of clemastine against promastigote parasites, using miltefosine and amphotericin B as controls. Clemastine fumarate demonstrated a slow-to-kill profile more akin to miltefosine (Figure 1D), an important consideration if this compound is to be clinically utilized.

### *Leishmania mexicana* Clemastine Fumarate Resistant Lines (ClemR) Demonstrate Similar Changes to Those Previously Seen in *Leishmania major* ClemR, Implicating Sphingolipid Biosynthesis

**Figure 2:**
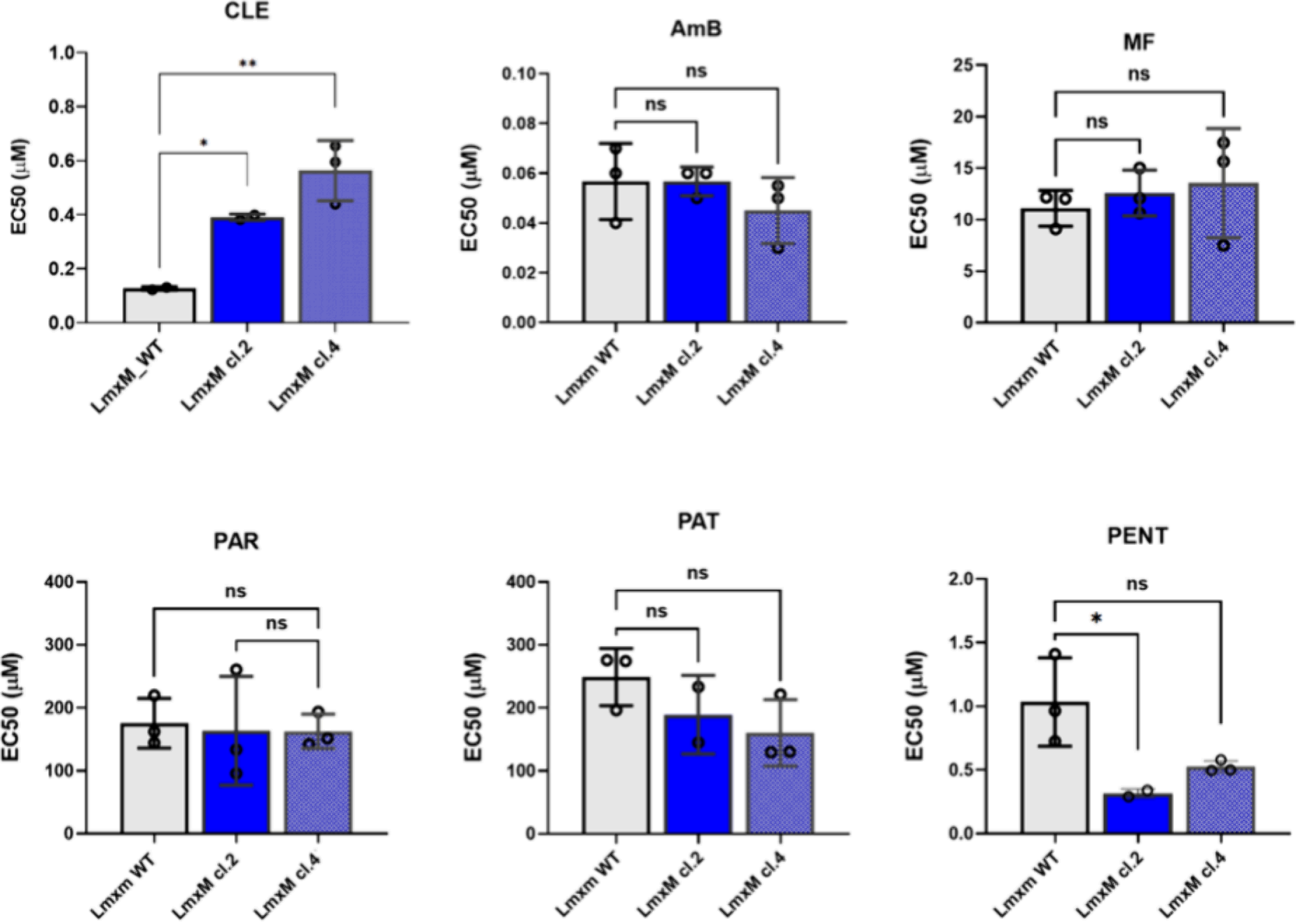
Analyses of the cross resistance (EC_50_) of selected clemastine fumarate resistant *Leishmania mexicana* against clinical anti-leishmanials. Clemastine resistant clones LmxM.cl.2 and LmxM.cl.4 of *L. mexicana* were tested against clemastine fumarate (CLE); amphotericin B (AmB); miltefosine (MF); paromomycin (PAR); potassium antimony tartrate (PAT); and pentamidine (PENT). EC_50_ values are the mean of at least three biological replicates with standard deviation (bars) and were processed using Prism software version 9.3.0. Statistically significant values (one-way ANOVAs with the Dunnett’s multiple comparison test, confidence interval *P*-value <0.05, 95%) are shown with stars: ns, nonsignificant; *P ≤ 0.05; **P ≤ 0.01.

In agreement with the enzyme inhibition data above, clemastine fumarate demonstrated efficacy against wild type *L. mexicana* promastigotes equivalent to that reported for *L. major*.^17^ Subsequently, resistant (ClemR) *L. mexicana* promastigote cell lines were selected using an increase of drug concentration in a stepwise manner (Figure S1), as previously described.^37,38^ Two clonal populations (LmxM.cl.2 and cl.4) from two independent clemastine-resistant cell lines (LmxM.cl.2 and cl.4) were derived by limiting dilution. To assess the stability of the resistant phenotype, the EC_50_ was assessed after parasites were cultured in the absence of drug for at least 15 passages. This rendered clonal lines that retained resistance to clemastine fumarate (Figure 2), LmxM.cl.2 (EC_50_ 0.39 ± 0.01 μM), and LmxM.cl.4 (EC_50_ 0.56 ± 0.10 μM) when compared to the parental line (EC_50_ 0.13 ± 0.01 μM). To test the possibility that the mode of resistance was shared with clinical anti-leishmanials, the selected and parental cell lines were screened with amphotericin B, miltefosine, paromomycin, potassium antimony tartrate, pentamidine and antimonials. No cross-resistance was observed demonstrating that the mechanism of resistance to clemastine fumarate was distinct. However, like clemastine-resistant *L. major*,^17^ the *L. mexicana* lines showed increased susceptibility to pentamidine (Figure 2).

**Figure 3:**
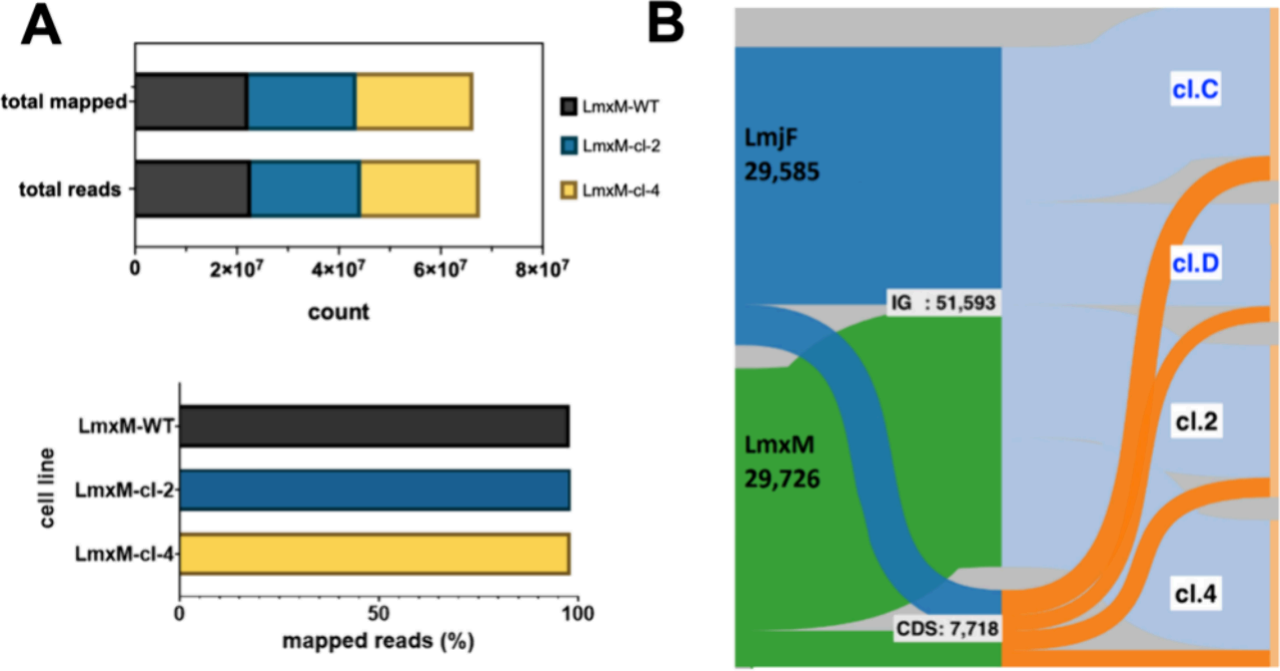
Whole genome sequencing analyses of the selected clemastine fumarate resistant *Leishmania mexicana*. (A) Bar charts showing total- and mapped-reads in *L. mexicana* wild type and two derived clemastine resistant (ClemR) clones LmxM.cl.2 and LmxM.cl.4. (B) Alluvial plot showing the distribution of SNPs in *L. mexicana* clones LmxM.cl.2 and LmxM.cl.4 (green) and their proportion across two categories, i.e. the coding (CDS, orange) and intergenic (IG, light blue) regions (*y-axis*) and how the distribution of SNPs changes across the mutants (*x-*axis) of both *L. mexicana* ClemR mutants (LmxM.cl.2 and LmxM.cl.4). Data from two *L. major* ClemR clones (dark blue; cl.C and cl.D) previously described^17^ are also shown for comparison.

**Figure 4:**
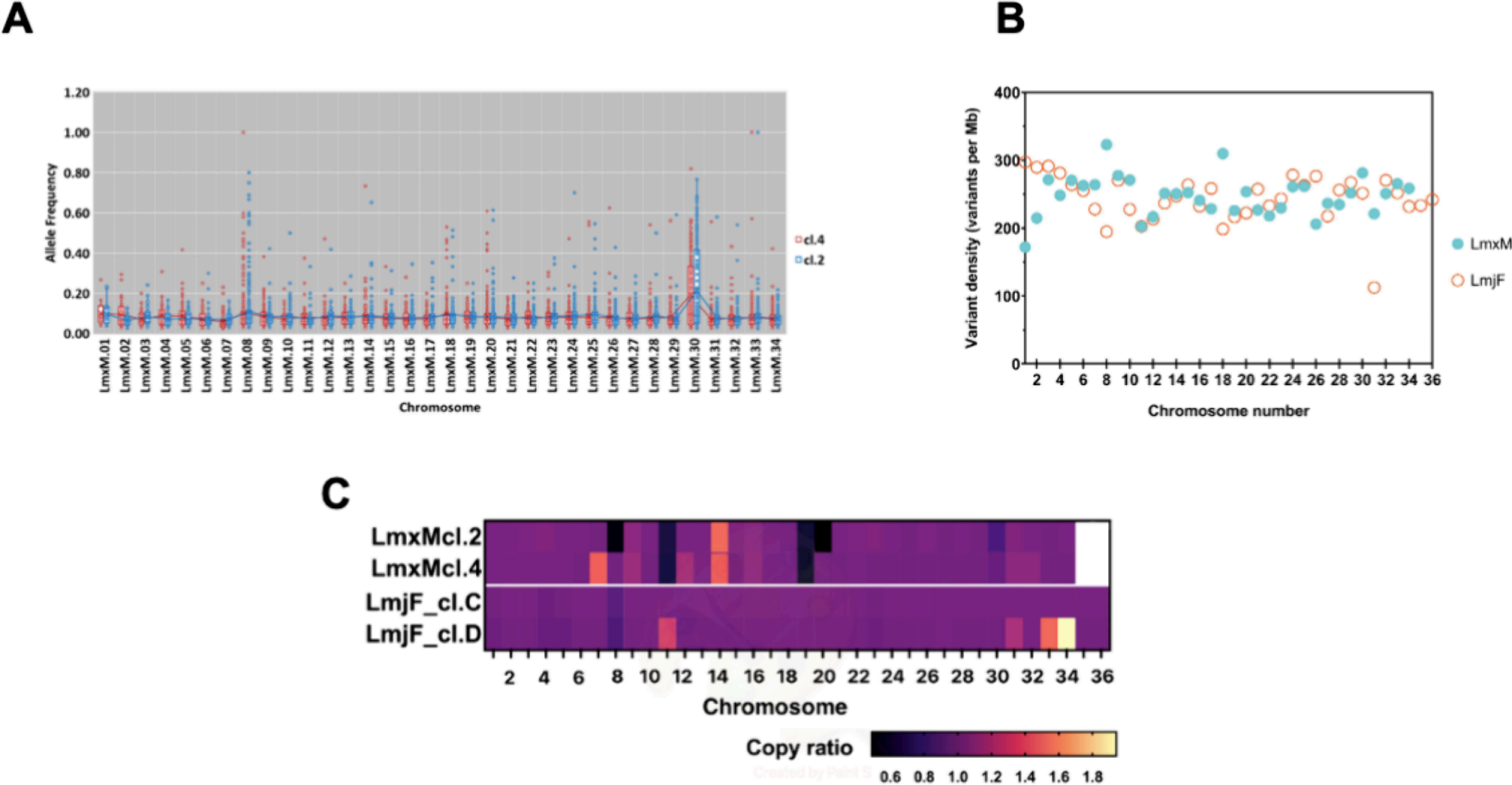
Impacts of clemastine fumarate on the *Leishmania mexicana* genome. (A) Allele frequency (AF) (*y-*axis) by chromosome (*x-*axis) of variants (SNPs) identified in *L. mexicana* ClemR clones LmxM.cl.2 (blue dots) and LmxM.cl.4 (red dots) in comparison with the parental wild type in the absence of drug pressure (black line). (B) Variant density (*y-*axis) by chromosome (*x-*axis) in both ClemR clones of *L. mexicana* (LmxM, blue filled circles). Data from two ClemR clones of *L. major* (LmjF, orange empty circles) described previously^17^ shown for comparison. (C) Heatmap showing copy-ratio changes (decrease: values < 1; increase: values > 1 and no change: values = 1) by chromosome (Chr). Values are the mean copy ratio of each Chr and were detected comparing the mutant clones with the corresponding copy number of the parent used as the baseline. Plot of copy-ratios was generated using Prism software version 9. See Table S4 for a detailed list of variants and CNV changes.

The plasticity of the genome in *Leishmania* spp. allows the parasites to adapt to different conditions including drug pressure.^39^ Therefore, we used whole genome sequencing (WGS) to identify SNPs, indels and copy number variations (CNVs) to identify genomic changes resulting from drug pressure with clemastine fumarate in selected clones LmxM.cl.2 and LmxM.cl.4, following a method previously described.^37,38^ In both the selected clones and the parental line, over 98% of the sequenced nucleotides were mapped to the reference genome (Figure 3A) and mean coverage was between 49 and 53×. The frequency of genomic variants within the coding sequences (CDS) and intergenic regions (IG) in LmxM.cl.2 and LmxM.cl.4 were comparable to that reported for two ClemR-*L. major* cell lines^17^ (Figure 3B and Table S1). Chromosomes (Chr) 8 and 30 showed the variants with the highest allele frequency (AF). In LmxM.cl4 Chr 8, a homozygous SNP (AF = 1) was found, while two others were present in Chr 30 from each (Figure 4A). Notably, Chr 8 and 20 (alongside 18, 30 and 31) showed a higher variant rate in *L. mexicana* than the *L. major* ClemR lines, which in general exhibited a relatively narrow range of variation between Chr (Figure 4B). This might be partly related to the linkage groups of *L. mexicana* Chr 8 and 20, where two fusion events occurred, one between Chr 8 and 28 and the second between Chr 20 and 36, and this resulted in a total of 34 Chr in *L. mexicana* contrasting of the 36 Chr found in *L. major*.^40^ Previous analyses in *L. major* identified multiple SNPs in genes associated with sphingolipid biosynthesis.^17^ Similarly, in *L. mexicana* LmxM.cl.4, missense SNPs in LmxM.33.3740 and LmxM.34.0320 (encoding subunits LCB1 and LCB2 respectively, forming serine palmitoyltransferase, the first, rate limiting step in sphingolipid biosynthesis) and LmxM.08.0200 (inositol phosphosphingolipid phospholipase C) were identified. Furthermore, sphingosine-1-phosphate phosphatase (putative, LmxM.31.2290) was the only gene in which nonsynonymous variants were found in both LmxM.cl.2 and LmxM.cl.4, and a similar variant in this gene was also found in clemastine fumarate resistant *L. major*.^17^ A list with the total number of variants in the sphingolipid biosynthetic pathway genes is provided (Table S2). In totality, this indicated that the *in vitro* response to drug pressure with the drug was partially conserved across *Leishmania* species with sphingolipid biosynthesis implicated in both *L. major* and *L. mexicana*.

As with the SNPs described above, aneuploidy contributes to diversity in *Leishmania* species^41^ and has been associated with drug resistance to various compounds.^17,37^ Perhaps reflecting this, a decreased copy number was found in LmxM.cl.2 Chr 8 and 20, while Chr 11 and 19 were decreased in both LmxM.cl.2 and LmxM.cl.4, with the copy-ratio suggesting the loss of one copy in each case. On the other hand, values indicated an increased copy number in LmxM.cl.4 Chr 7 and in Chr 14 from both clones (Figure 4C and Figure S2). From these, only Chr 8 harbors a gene known to encode a protein of the sphingolipid biosynthetic pathway (LmxM.08.0200, inositol phosphosphingolipid phospholipase C [ISCL]). Significantly, a decrease in the same Chr was identified in both clemastine fumarate resistant *L. major* clones analyzed,^17^ although the fact that only one of the *L. mexicana* derived clones showed the variation limits the interpretation of this finding. However, collectively, these results supported the hypothesis that the genome plays a role in the development of resistance to clemastine fumarate, with several genomic changes, i.e., SNPs and CNVs, indicating a common mode of action implicating sphingolipid biosynthesis (Figure S3).

### Clemastine Fumarate Treatment of *Leishmania mexicana* Leads to Lipidomic Changes Which Indicate Disruption of Inositol Phosphorylceramide Synthase (*Lmx*IPCS) activity

Using untargeted LC-MS metabolomics and lipidomics, we probed the impact of clemastine fumarate on cellular processes in wild type *L. mexicana* promastigotes. Principal Component Analysis (PCA) showed the reproducibility of the methodology (Figure S4A) and extensive metabolic changes occurred on treatment, although clear, individual target could not be readily identified (Figure S4B). The untargeted mass spectrometry approach was able to identify clemastine and metabolites of the drug, and comparable with other eukaryotes,^42^ clemastine was biotransformed into three metabolites present at high concentrations (dihydroclemastine, hydroxyclemastine and norclemastine) in both *L. mexicana* and *L. major*. The abundance of each was different between these species (Figure S5A).

**Figure 5:**
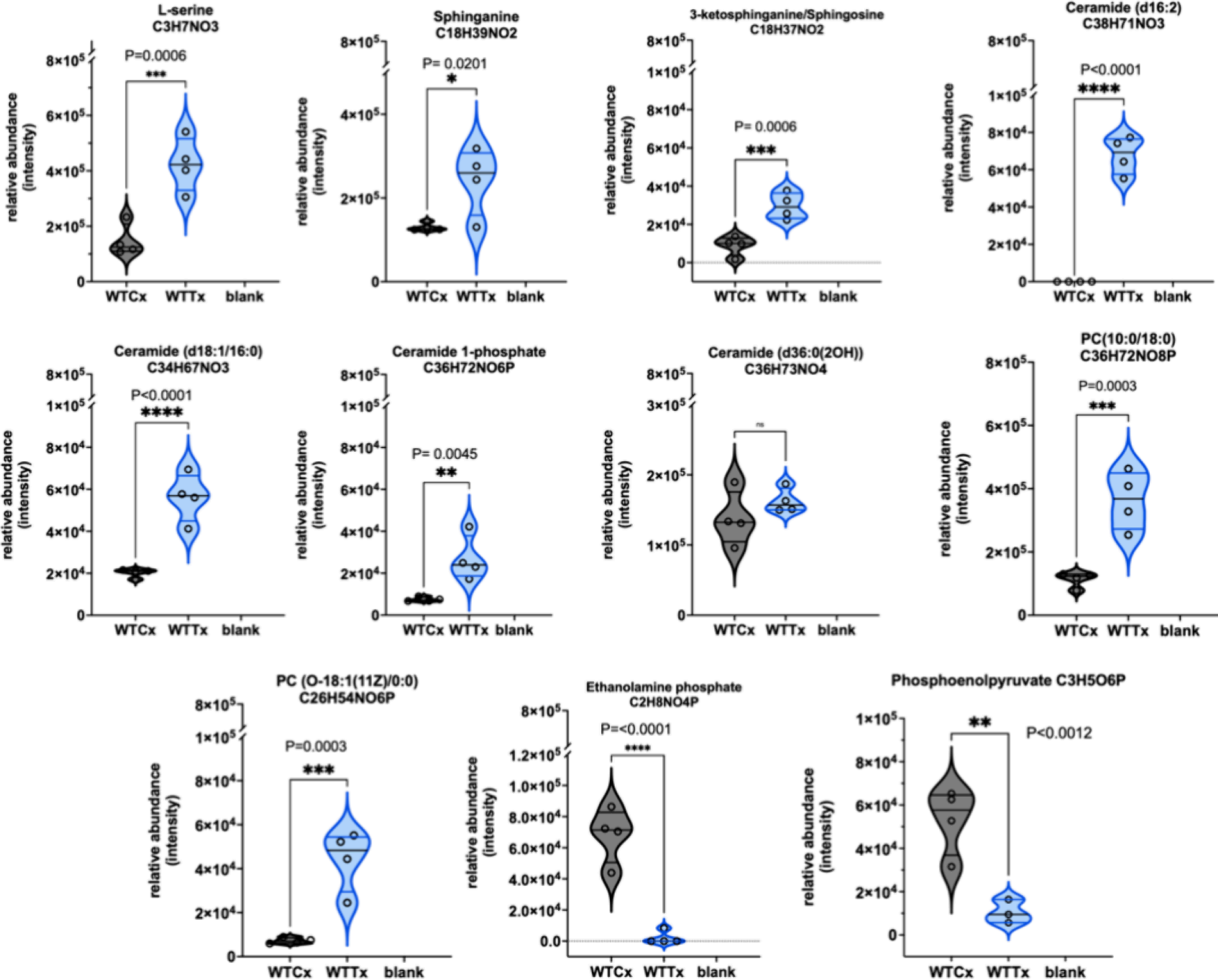
Relative abundance (**y*-*axis) of metabolites identified in *Leishmania mexicana* after clemastine fumarate (10 μM) exposure for 12 h. Biological replicates (*n* = 4) of treated (WTTx) and untreated parasites (WTCx) (*x-*axis) were analyzed with LCMS and processed using multivariate data analysis with PiMP pipeline.^70^ The Benjamini-Hochberg procedure adjusted raw *P*-values (*q*-values) < 0.05 for ANOVA.

Lipidomic analyses demonstrated significant disruption of lipid metabolism with large increases in the relative abundance of ceramide species after clemastine fumarate treatment (Figure S5B). Further focused analyses showed that wild type *L. mexicana* responded in a manner closely resembling that reported for *L. major*,^17^ with statistically significant increases in sphinganine, 3-ketosphinganine (sphingosine), multiple ceramide species, and ceramide-1-phosphate (Figure 5). All of these could rationally be associated with inhibition of IPCS^17^ and influenced by the sphingolipid biosynthetic SNPs detected in resistant *L. mexicana* lines as illustrated in Figure S3. For example as noted in *L. major*,^17^ a decrease in the expression level of the phosphosphingolipid phospholipase C (ISCL; Figure S3-4 and Table S2) could lead to the accumulation of IPC and, perhaps, clemastine fumarate resistance.^17^ Similarly, for example, mutations in the serine palmitoyltransferase (SPT; Figure S3-1 and Table S2) and ceramide desaturases (CerD; Figure S3-3 and Table S2) could reduce the levels of sphinganine and ceramide respectively, perhaps eliciting a protective effect as these metabolites are seen to rise on clemastine fumarate treatment (Figure 5). In addition, of course, mutations in the putative target itself, IPCS (Figure S3-5 and Table S2), could influence resistance.

Furthermore, and again reflecting the *L. major* data,^17^ phosphatidylcholine (PC) levels were increased on clemastine fumarate treatment. However, in a deviation from the *L. major* response, l-serine levels were increased, and ethanolamine phosphate levels decreased to near zero in treated *L. mexicana* (Figure 5). Both of these showed no response to clemastine fumarate in *L. major*,^17^ perhaps indicating a more complex mode-of-action for this anti-leishmanial compound.

To investigate this further we undertook thermal proteomic profiling (TPP) in wild type *L. mexicana* to identify proteins that bind to clemastine fumarate. A comprehensive abundance pattern of soluble proteins was observed following extraction from promastigote parasites, highlighting distinct thermal shifts differences between treated samples and controls (Figure S6). Notably, 41 of these proteins exhibited significant shifts in clemastine fumarate-induced melting temperature (Δ*T*_m_ ≥ 4; Table S5), underlining the complexity of the mode-of-action of clemastine fumarate. None of the hits directly supported inhibition of sphingolipid biosynthesis, perhaps not surprisingly given the membrane bound nature of the enzymes associated with this.^19−23^ However, potential clemastine fumarate binding proteins included numerous glycosomal and mitochondrial enzymes: succinate dehydrogenase, 2-oxoglutarate dehydrogenase subunit, NADH-dependent fumarate reductase, fumarate and pyruvate phosphate dikinase. Intriguingly, a substantial reduction in the tricarboxylic acid (TCA) cycle was evident in our metabolomic data set on clemastine fumarate treatment (Table S6). Furthermore, treatment also led to a 2.4-fold depletion of phosphoenolpyruvate (PEP) in *L. mexicana* (Figure 5), and this reduction was more pronounced in *L. major* (3.2-fold, *P* < 0.0001), where two TCA metabolites, oxoglutarate (*P* < 0.0060) and citrate (*P* < 0.0197), were also significantly decreased (Figure S7). PEP is fermented to succinate via the glycosomal succinate shunt to replenish ATP and NAD^+^ consumed in the glycolytic pathway.^43^ In *L. mexicana*, seemingly unlike other trypanosomatids, the TCA cycle assumes significant roles in anabolic pathways, and these glycosomal and mitochondrial enzymes are closely intertwined.^43^

However, given that the fumarate salt of clemastine was utilized in our analyses, we could not exclude the possibility that the TCA metabolite fumarate had a direct effect on this process and, by implication the glycosomal succinate shunt. Indeed, fumarate levels increased significantly in treated *L. major* (Figure S7), although this metabolite was not observed in the *L. mexicana* data set (data not shown). In summary, while clemastine fumarate treatment clearly disrupts *Lmx*IPCS functionality, other effects are noted; however, these are not clearly defined and will require further investigation in the future.

### Deletion of the *Leishmania mexicana* Inositol Phosphorylceramide Synthase (*Lmx*IPCS) Leads to Loss of Inositol Phosphorylceramide

**Figure 6:**
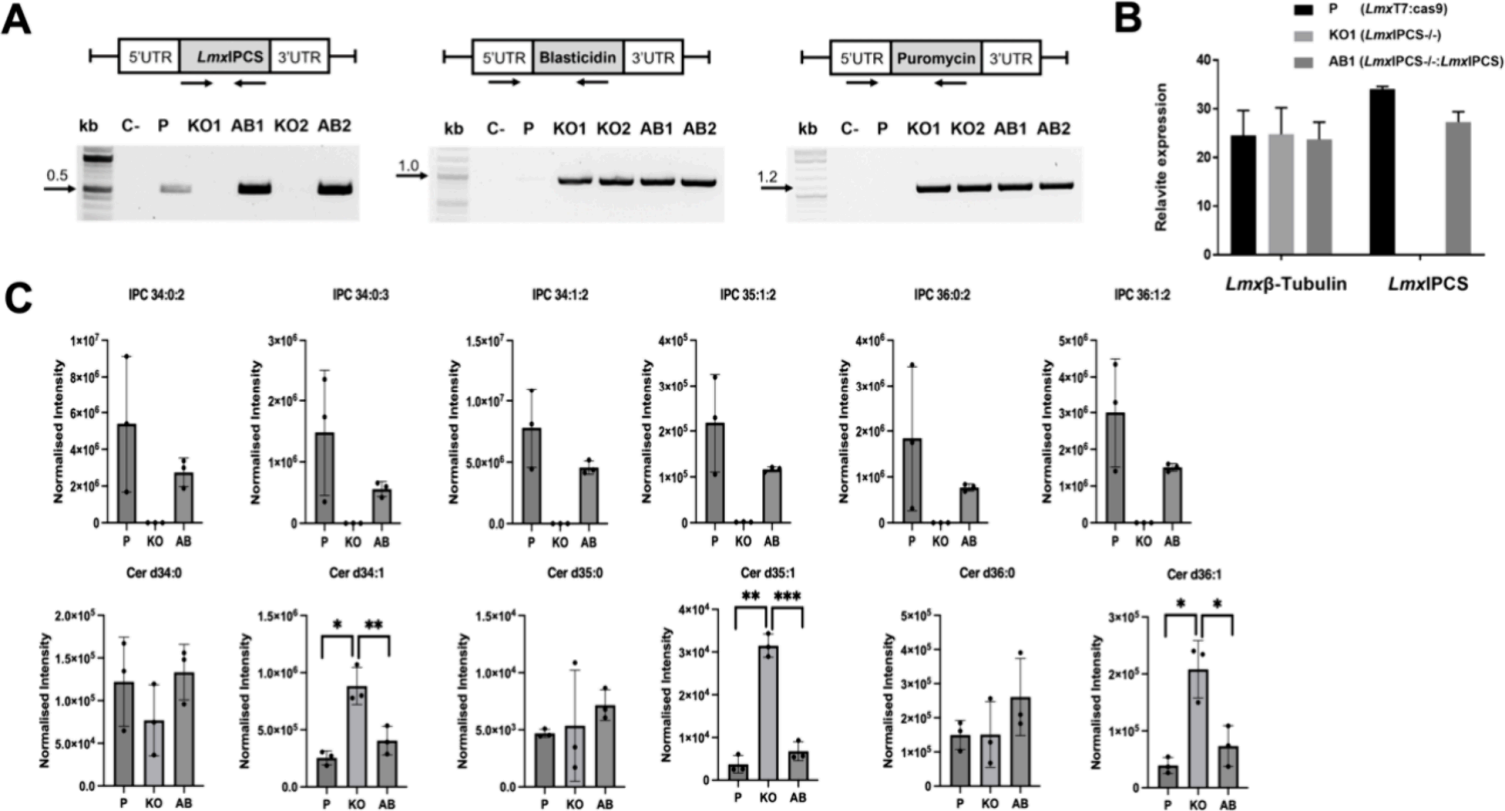
Genetic deletion of the *Leishmania mexicana* inositol phosphorylceramide synthase (*Lmx*IPCS) leads to loss of IPC synthesis. (A) Cell lines were generated by deleting (KO; *Lmx*IPCS–/−) and restoring (AB; *Lmx*IPCS–/–:*Lmx*IPCS) the *Lmx*IPCS utilizing CRISPR/Cas9 technology. Two KO clones (KO1 and KO2) were tested to ensure complete removal of the target gene before the restoration of *Lmx*IPCS (AB1 and AB2). The success of transgenic generation was confirmed through PCR, using primer sets that anneal to the open reading frame or resistance markers. The parental line (P) was used as a positive control, and a negative control (C−) was included with no DNA added. (B) RT-qPCR was performed to assess the expression level of the gene in the samples from P (*Lmx*T7:Cas9), KO (KO1 *Lmx*IPCS–/−) and AB (AB1 *Lmx*IPCS–/–:*Lmx*IPCS), with the β-tubulin gene used as reference. (C) LC-MS analyses confirmed the loss and return of IPC in KO (KO1) and AB (AB1), and the associated loss and return of ceramide species. Differences between samples (P vs AB; P vs KO; KO vs AB) were evaluated using paired *t* tests in Prism software version 9.3.0. Statistically significant values are shown with stars: *p ≤ 0.05; **p ≤ 0.01; ****p ≤ 0.0001. Changes in cardiolipin species (Figure S9) are also provided.

To evaluate the drug target status of *Lmx*IPCS further, knockout cell line clones (*Lmx*IPCS–/–; KO1 and KO2) and their respective complemented or add-backs lines (*Lmx*IPCS–/–:*Lmx*IPCS; AB1 and AB2) were generated by CRISPR-Cas9 mediated deletion of the gene of interest and its subsequent reintroduction into the β-tubulin locus.^37^ The successful generation of these transgenic cell was confirmed using a PCR diagnostic approach (Figure 6A). Additionally, quantitative PCR confirmed that the gene of interest is expressed in the parental line (*Lmx*T7:Cas9) and the add-back *Lmx*IPCS–/–:*Lmx*IPCS, whereas *Lmx*IPCS–/– shows no expression (Figure 6B). To assess whether the synthesis of IPC is solely dependent on *Lmx*IPCS, promastigotes were metabolically labeled with C_6_-NBD-ceramide before separation and analysis by TLC. This indicated that IPC production was entirely dependent upon the gene target (Figure S8). LC-MS lipidomic analyses unequivocally confirmed the loss of IPC in *Lmx*IPCS–/– and, importantly, its regain in *Lmx*IPCS–/–:*Lmx*IPCS (Figure 6C). However, all six detected IPC species failed to return to parental levels, although these observations were not statistically significant. Furthermore, the analyses also revealed that, as expected of the enzyme substrate, several ceramide species accumulated in the *Lmx*IPCS–/– samples to statistically significant levels compared to the parental line and *Lmx*IPCS–/–:*Lmx*IPCS: Cer d34:1, d35:1, and d36:1—the direct substrates for the production of IPC 34:1:2, 35:1:2, and 36:1:2 (Figure 6C).

### Loss of *Leishmania mexicana* Inositol Phosphorylceramide Synthase (*Lmx*IPCS) Is Tolerated; However Inositol Phosphate Synthesis and Drug Sensitivity Is Altered

**Figure 7:**
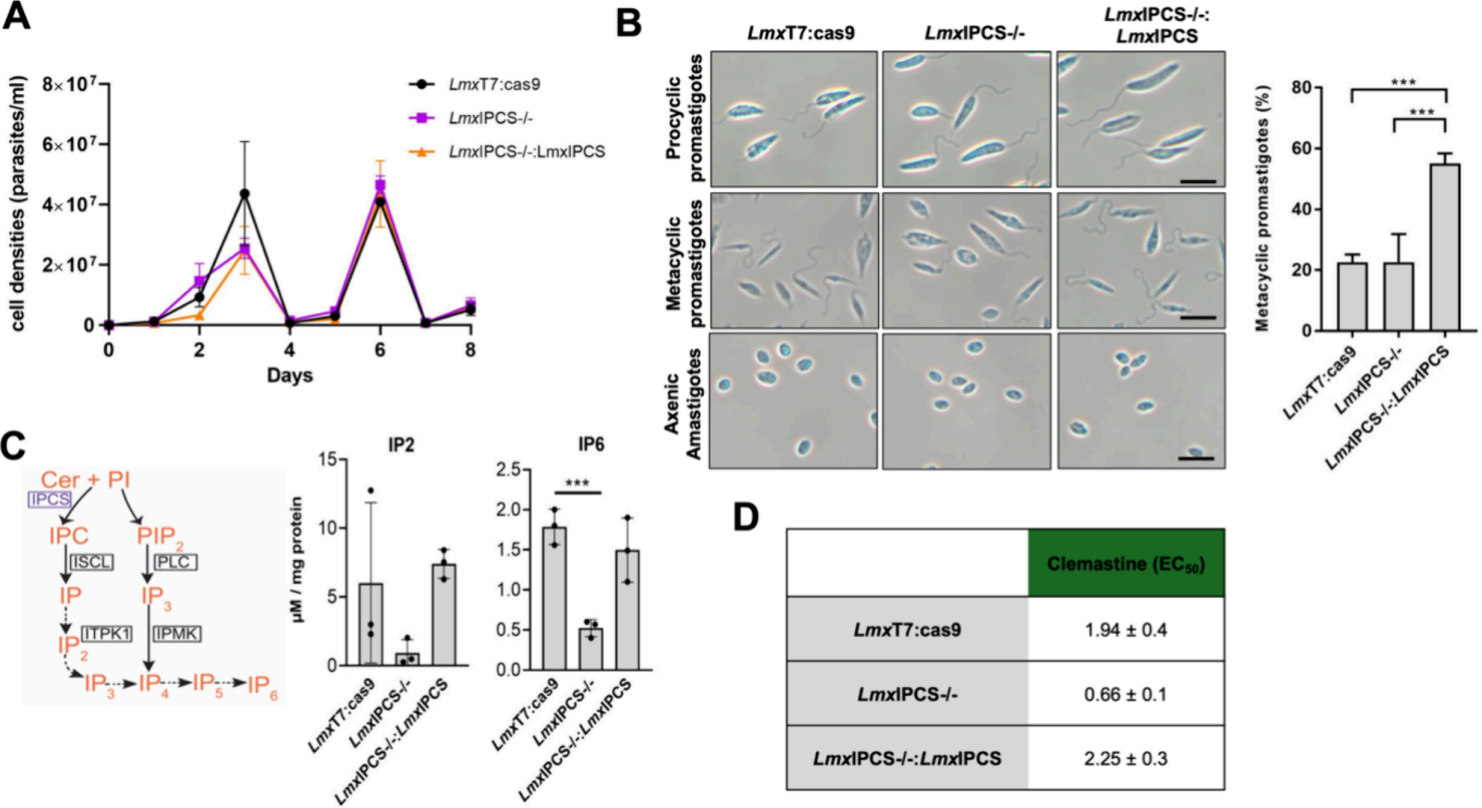
*In vitro* phenotypic analyses demonstrated loss of inositol phosphorylceramide synthase (*Lmx*IPCS) is associated with changes in soluble inositol polyphosphates and clemastine fumarate efficacy in *Leishmania mexicana*. (A) The growth of procyclic promastigote transgenic cell lines (*Lmx*IPCS–/– and *Lmx*IPCS–/–:*Lmx*IPCS) showed no discernible difference to the parental (*Lmx*T7:Cas9). The sawtooth pattern is due to the cells being subcultured every 3 days at 1 × 10^6^/mL to maintain log-phase growth. (B) Morphological observations (left-hand panel) showed that promastigotes and axenic amastigotes displayed normal morphological phenotypes in the absence of IPCS (*Lmx*IPCS–/−) and its re-expression (*Lmx*IPCS–/–:*Lmx*IPCS). Scale: 10 μm. However (right-hand panel), detailed analyses of the flagella:body length ratio using ImageJ demonstrated *Lmx*IPCS–/–:*Lmx*IPCS to have statistically significant enhanced metacyclogenesis compared to both the parental (*Lmx*T7:Cas9) and *Lmx*IPCS–/– lines. Unpaired *t* test, *** *p* value <0.001. (C) Schematic depiction of the soluble IPC-dependent pathway identified in this study (left-hand panel). Enzymes in boxes possess gene sequences annotated in the *L. mexicana* genome. The ITPK1 (Gene accession number: LmxM.24.1930) could be the kinase acting on soluble IP species which stem from IPC (dotted arrows). Although the PLC, inositol multiphosphate kinase (IPMK), has also been identified in the genome its contribution to the whole IP pool has not yet been addressed. Quantitation of IP_2_ and IP_6_ content (right-hand panel) was performed by LC-MS/MS using inositol 1,4-bisphosphate (IP_2_) and phytic acid (IP_6_) as standards. Values plotted are mean ± SD from biological triplicates. Values were calculated from standard curves using known concentrations of each analyte and normalized to total protein content. Comparisons were made using unpaired *t* test, *** *p* value <0.001. Ceramide: Cer, Phosphatidylinositol: PI, Phosphatidylinositol bisphosphate (PIP_2_). (D) Dose–response assays using clemastine fumarate revealed that *Lmx*IPCS–/– had increased sensitivity which was restored to parental (*Lmx*T7:Cas9) levels in the add back (*Lmx*IPCS–/–:*Lmx*IPCS). EC_50_ values shown in μM with 95% confidence intervals.

The importance of the *Lmx*IPCS enzyme in *L. mexicana* was evaluated in cell culture, focusing on its impact on replication, morphology, inositol phosphate (IP) metabolism, and clemastine sensitivity. Procyclic transgenic parasites, *Lmx*IPCS–/– (KO1, Figure 6A) and add-back *Lmx*IPCS–/–:*Lmx*IPCS (AB1, Figure 6A), exhibited growth patterns comparable to the parental control, *Lmx*T7:Cas9 (Figure 7A). This suggested that loss of *Lmx*IPCS is not crucial for procyclic promastigote fitness. However, while no substantial differences were observed in the morphology of the procyclic forms of the cell lines, a notable disparity in the percentage of metacyclic promastigotes was observed (Figure 7B). Metacyclogenesis is accompanied by several morphological changes, and while procyclics possess flagella of approximately one body length, in metacyclic forms the relative flagella length is longer.^44^ Using this as a marker it was observed that 55% of the add-back parasites (*Lmx*IPCS–/–:*Lmx*IPCS) were metacyclic compared to only 23% of the parental line (*Lmx*T7:Cas9) and the parasites lacking *Lmx*IPCS (*Lmx*IPCS–/−). Notably, these differences did not interfere in *in vitro* differentiation into axenic amastigote forms (Figure 7B).

The complex lifecycle, including differentiation and pathology, of trypanosomatids such as *Leishmania* species is subject to a network of signaling and regulation that include inositol phosphates (IPs).^45^ Recent studies have demonstrated the presence of a phospholipase C (PLC)-independent pathway leading toward formation of IPs from glucose and IPC precursors.^46,47^ In this pathway, glucose 6-phosphate can isomerize to inositol 3-monophosphate (I_3_P), and IPC can be cleaved to release inositol 1-phosphate (I_1_P). Both these species can be further phosphorylated by an archaeal inositol tetrakisphosphate kinase (ITPK1) that restores IP_6_ levels in a Δ*plc* background yeast.^46^

Considering the abundance of IPC in *Leishmania* parasites (15% of phospholipids^48^), and by analogy with yeast,^46^ this sphingolipid may also be involved in IP synthesis via phosphosphingolipid phospholipase C-like driven cleavage to yield I_1_P (ISCL; Figure 7C).^49,50^ Notably, *Lmx*ISCL (LmxM.08.0200) was mutated in both clemastine fumarate resistant *L. mexicana* clones (Table S2), with a similar outcome in *L. major*.^17^ In light of these observations, we examined whether the pool of IPs is altered in response to changes in IPC content. *Lmx*IPCS–/– produced considerably less IP_2_ and IP_6_ than parental, with levels restored after reconstitution of the enzyme IPCS and IPC (*Lmx*IPCS–/–:*Lmx*IPCS; Figure 7C). These data reflected the global role of this enzyme and its product in *Leishmania* species, perhaps underwriting the phenotypes observed in the knockout analyses.

IPCS has been identified as a target for clemastine fumarate in *L. major*.^17^ Here we demonstrated that this compound has equivalent activity at a cellular and enzyme level in *L. mexicana* (Figures 1 and 2). Utilizing the generated IPCS KO (*Lmx*IPCS–/−) and add-back (*Lmx*IPCS–/–:*Lmx*IPCS) cell lines we further investigated this proposed mode-of-action, theorizing that loss of the target would confer resistance to clemastine fumarate. Dose–response assays revealed an EC_50_ of 1.94 ± 0.4 μM against the procyclic *L. mexicana* parental line (*Lmx*T7:Cas9), a higher value than we found for the wild type parasites (Figure 2) and an effect we have noted with other drugs and compounds (data not shown). However, and surprisingly, the absence of IPCS (*Lmx*IPCS–/−) rendered the parasites significantly more sensitive to clemastine fumarate, a phenotype that was reversed upon restoration of the gene (*Lmx*IPCS–/–:*Lmx*IPCS) (Figure 7D). In contrast, and in alignment with the sensitivity of the characterized sphingolipid-free *L. major* line, *Lmj*LCB2–/–,^51,52^ miltefosine sensitivity is reversibly decreased in *Lmx*IPCS–/–, while amphotericin B sensitivity is reversibly increased (Table S3). Interestingly, these data do not support recently reported findings showing that IPCS KO alone in *L. major* does not alter sensitivity to amphotericin B.^53^

### *Leishmania mexicana* Inositol Phosphorylceramide Synthase (*Lmx*IPCS) Is Important for Parasite Pathogenicity

**Figure 8:**
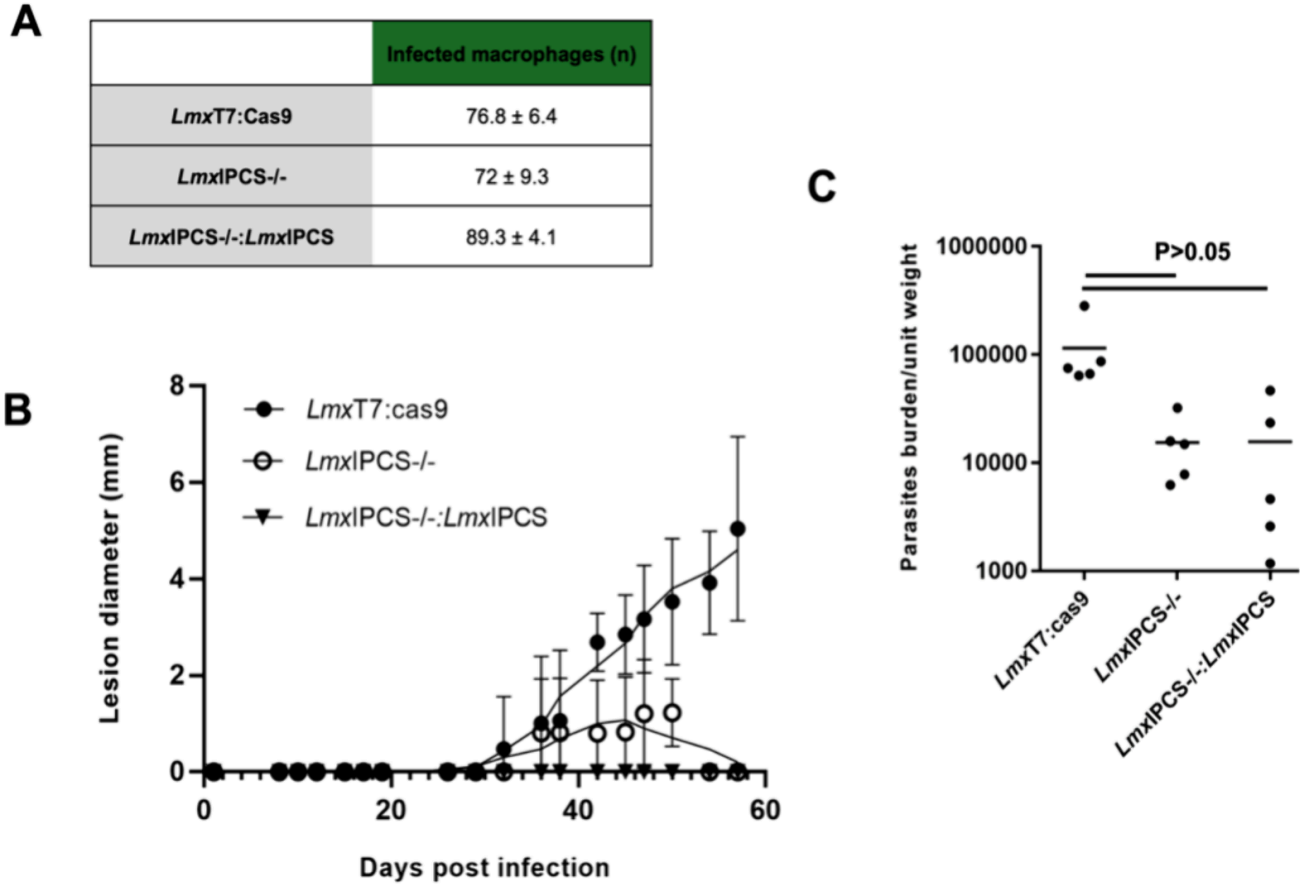
The loss of inositol phosphorylceramide synthase (*Lmx*IPCS) is associated with an *in vivo* pathogenic deficit in *Leishmania mexicana*. (A) Infected macrophages were counted at 72-h postinvasion. No significant differences were observed among the three cell lines under study. Data expressed as mean ± SD. (B) The size of the lesions caused by parasite infection in mice was measured over an 8-week period. Lesions caused by the parental cell line (*Lmx*T7:Cas9, black circle) increased steadily throughout the weeks. Lesions caused by the IPCS mutant (*Lmx*IPCS–/–, white circle) exhibited slower growth and eventually started to heal. Mice infected with the add-back cell line (*Lmx*IPCS–/–: *Lmx*IPCS, black triangle) did not develop lesions. (C) Parasite load analysis revealed the presence of parasites in the lesion area of all infected mice. Mice infected with the parental cell line (*Lmx*T7:Cas9) exhibited a higher parasite load compared to *Lmx*IPCS–/– and *Lmx*IPCS–/–: *Lmx*IPCS, although comparisons using unpaired *t* test showed no statistically significantly differences between the parasite lines.

The infectivity of all three lines (parental *Lmx*T7:Cas9; KO *Lmx*IPCS–/–; and add-back *Lmx*IPCS–/–:*Lmx*IPCS) was assessed *in vitro* using a stationary-phase promastigote and mouse peritoneal macrophage (PEM) infection assay platform (Figure 8A). No significant differences were observed, and subsequently the pathogenicity of the parasites was assessed in a murine model over an 8-week period. Infection with *Lmx*T7:Cas9 and *Lmx*IPCS–/– led to lesions developing 30 days postinfection. The lesion sizes in mice infected with *Lmx*T7:Cas9 showed a steady increase until day 60 post-infection. Conversely, in mice infected with *Lmx*IPCS–/– the lesion size increased slowly before eventually healing completely. Notably, mice infected with *Lmx*IPCS–/–:*Lmx*IPCS did not develop any detectable lesions (Figure 8B). To further understand this phenomena, *Lmx*T7:Cas9, *Lmx*IPCS–/– and *Lmx*IPCS–/–:*Lmx*IPCS parasites were harvested from the lesion area at the end of the experiment (day 60 postinfection) and quantified. The results revealed the presence of parasites in all cases, although the number of parasites in the lesion area was 10 times higher (*P* > 0.05) in mice infected with *Lmx*T7:Cas9 compared to both *Lmx*IPCS–/– and *Lmx*IPCS–/–:*Lmx*IPCS (Figure 8C). The failure to fully recover IPC levels (Figure 6C), although not statistically significantly, may be behind the lack of *Lmx*IPCS–/–:*Lmx*IPCS pathogenicity. Furthermore, cardiolipin levels which were reduced in *Lmx*IPCS–/– did not recover to parental (*Lmx*T7:Cas9) levels in the add back, with five of the 11 species detected statistically significantly lower in *Lmx*IPCS–/–:*Lmx*IPCS compared to *Lmx*T7:Cas9 (Figure S9). Cardiolipins, unique phospholipids which are localized and synthesized in the inner mitochondrial membrane, have been previously identified in *Leishmania* species^54,55^ and have been demonstrated to be essential in *Trypanosoma brucei*.^56^

## Discussion

In light of the recognized urgent need for new therapies for the Neglected Tropical Disease leishmaniasis, the identification of clemastine fumarate, an over-the-counter antihistamine, as a potential drug candidate is important.^17,18^ The polypharmacological nature of clemastine, which includes its inhibition of inositol phosphorylceramide synthase (IPCS), alongside the absence of cross resistance against the clinical antileishmanials positions this orphan drug as a promising candidate for further exploration.

In this study we focused on *L. mexicana*, whose genetic tractability and *in vitro* axenic lifecycle facilitate in depth analyses of the role of *Lmx*IPCS in the parasite and infection, as well as further understanding of the mode of action of clemastine fumarate. Comparative analyses across *Leishmania* species, *L. mexicana* and *L. major*, uncovered both similarities and differences in metabolic disturbances and mutations induced by clemastine. Whole genome analyses identified genetic changes associated with clemastine fumarate resistance, providing critical insight into the mode of action and potential resistance mechanisms, and extensive exploration of metabolomic and lipidomic changes in response to clemastine fumarate shed further light on the broader impact of the compound on cellular processes. This holistic approach is invaluable for uncovering potential secondary effects and understanding the complexity of the *Leishmania* response, and *L. mexicana* (data presented here) and *L. major*(17) clearly share a largely common response to clemastine fumarate with a disruption of sphingolipid biosynthesis. However, differences are also evident as shown by the reverse response of ethanolamine phosphate (Figure 5). However, the common depletion of phosphoenolpyruvate both in *L. mexicana* (Figure 5) and *L. major*(17) point toward clemastine fumarate mediated TCA cycle disruption, further complicating our understanding of the mode of action. This observation is supported by data from thermal proteomic profiling (TPP; Figure S6 and Table S5) which indicated that proteins in respiration are directly impacted, although whether directly by clemastine or its salt fumarate is unclear given that the latter is a TCA metabolite.

This possible targeting of respiration may also be related to the increased susceptibility to the clinical antileishmanial pentamidine observed in all four clemastine fumarate resistant mutants of *L. mexicana* and *L. major*.^17^ Pentamidine has shown inhibition of the active transport system and inhibition of mitochondrial topoisomerase II leading to parasitic death in *L. donovani* and *L. amazonensis*.^57^ An allied study showed that a reduced pentamidine uptake in *L. mexicana* was related to a decreased mitochondrial membrane potential.^58^ Likewise, data from *L. major* have suggested that changes in mitochondrial membrane potential^59^ in addition to lipid remodelling^17,18^ underlie pentamidine hypersensitivity.

The ablation of *Lmx*IPCS was easily achieved indicating a non-essential function *in vitro* (*Lmx*IPCS–/–; Figure 6). These cells lacked IPC but were viable and grew normally in cell culture (Figure 7). In addition, and perhaps in keeping with the initial proposed mode of action, loss of IPCS (*Lmx*IPCS–/−) also had a major and reversible impact on clemastine fumarate sensitivity (Figure 7). However, rather than the expected resistance phenotype due to the loss of the target, the *Lmx*IPCS–/– parasites were approximately 3-fold more sensitive. This adds further weight to a more complex mode of action for clemastine fumarate, although it does place sphingolipid biosynthesis at the center of this. Indeed, looking further at inositol lipids in the transgenic parasites we demonstrated, for the first time, that in addition to the canonical PLC-dependent route, *Leishmania* parasites have evolved to use IPC sphingolipids as precursors for the inositol phosphate (IP) biosynthetic machinery. Given the roles of IPs in signal transduction this effect could have a major impact on *L. mexicana* biology, drug sensitivity and pathogenicity. The potential core roles of IPCS, IPC and IPs are supported by the fitness cost evidenced in *in vivo* (Figure 8). These data do not reflect those recently observed for similar analyses in *L. major* where KO of the orthologous gene by conventional homologous recombination and drug section leads to hyperinfectivity in a very similar murine cutaneous disease model.^33^ This could be explained by species specific differences, or alternatively, as we have recently reported, this technology can lead to untargeted compensatory deletions which mask the true fitness cost of deletions.^38^ Unfortunately, restoration of gene function in *L. mexicana* (*Lmx*IPCS–/–:*Lmx*IPCS; Figure 8) did not recover the parental phenotype. This correlated with the failure of the add back to recover parental levels of IPC (Figure 6) and cardiolipid species (Figure S9). Furthermore, our very recent parallel study of the IPCS in the related kinetoplastid parasite *Trypanosome cruzi* showed the exact same phenotype with lipid changes beyond the sphingolipids postulated to be behind this.^60^

## Conclusion

In totality, the reported findings contribute not only to the specific understanding of clemastine fumarate and IPCS in *Leishmania* species but also to the broader field of Neglected Tropical Diseases. The detailed insights into the molecular and genetic aspects of drug response, coupled with *in vivo* observations, pave the way for informed drug development strategies. The study’s holistic approach positions it as a pivotal step toward addressing the urgent need for improved treatments for leishmaniasis and, potentially, other Neglected Tropical Diseases. However, clearly the complexity of the outcomes with a polypharmacologic agent such as clemastine fumarate make data interpretation and identification of a mode of action challenging. That said, future research can build upon this study to develop more targeted, less toxic, and more resilient antiprotozoals.

## Methods

### Animals and Ethics Statement

All animal work was carried out under a UK Home Office project license according to the Animal (Scientific Procedures) Act 1986 and the European Directive 2010/63/EU. The project license (PPL P1651724) was reviewed by the University of York Animal Welfare and Ethical Review Board prior to submission and consequent approval by the U.K. Home Office.

### *Leishmania* Cell Culture

Procyclic promastigote *L. mexicana* (MNYC/BZ/62/M379 strain), *L. majo*r (MHOM/IL/80/Friedlin strain), and transgenic *L. mexicana* cell lines were cultivated in Schneider’s medium pH 7.0 (Gibco) supplemented with 15% heat inactivated Foetal Bovine Serum (FBS; Gibco) and 1% Penicillin-Streptomycin (Gibco). Cultures were maintained at 26 °C. *L. mexicana* (MNYC/BZ/62/M379 strain) expressing Cas9 and T7 RNA polymerase (Parental; LmxT7:Cas9),^61^ and all transgenic cell lines generated were maintained as described above in the presence of 32 μg/mL hygromycin B and 50 μg/mL nourseothricin sulfate (*Lmx*T7:Cas9). *Lmx*IPCS knockout (KO, *Lmx*IPCS–/−) lines were maintained in the presence of 20 μg/mL puromycin dihydrochloride and 5 μg/mL blasticidin S hydrochloride, and the add-back (AB, *Lmx*IPCS–/–:*Lmx*IPCS) lines were supplemented with 40 μg/mL Geneticin disulfate (G-418). The differentiation to metacyclic promastigotes and axenic amastigotes was induced by culturing parasites in Schneider’s medium pH 5.5, 20% FBS at 26 and 32 °C, respectively as previously described.^62^

### Rate of Kill

Promastigote NanoLuc-PEST *L. mexicana*(63) at 1 × 10^6^ /mL were treated in triplicate with 1:2 serial dilutions of clemastine fumarate, amphotericin B, miltefosine and vehicle control in 96-well plates (Fisher Scientific). Clemastine fumarate and miltefosine assays each constituted two biological replicates with three technical replicates in each (*n* = 6); the amphotericin B assay constituted a single biological replicate with three technical replicates (*n* = 3). Plates were sealed and incubated at 26 °C before reading the bioluminescence signal at 2.5, 17, and 24 h post treatment using the Nano-Glo Luciferase Assay Kit (Promega) in white 96-well plates (Greiner, UK) according to the manufacturer’s instructions. Subsequently, the bioluminescence signal was quantified using the GloMax Multi Detection System (Promega) and normalized with respect to positive (amphotericin B) and negative (vehicle, DMSO) controls (eq 1). Relative bioluminescence was fitted to a four-parameter log–logistic model using the ‘log(inhibitor) vs response -- Variable slope’ function of GraphPad Prism (version 10.0.2).
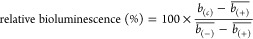
1where *b*_(*c*)_ = bioluminescence at a given compound concentration (*c*);  = mean bioluminescence of the positive control (amphotericin B);  = mean bioluminescence of the negative control (vehicle, DMSO).

### Generation and Analyses *Leishmania mexicana* Clemastine Resistance

Each *L. mexicana* independent line and individual clones were selected for resistance to clemastine fumarate as described in our previous work.^17^ Whole Genome Sequencing (WGS) of both clemastine resistant *L. mexicana* clones (LmxM.cl.2 and LmxM.cl.4) was performed following the bioinformatics pipeline previously described.^17^ Raw sequence data were deposited at the European Nucleotide Archive (ENA) under project number PRJNA665266.

### LC-MS Metabolomic and Lipidomic Analyses of the *Leishmania mexicana* Response to Clemastine Fumarate

Metabolomic and lipidomic extractions were performed on wild type *L. mexicana*, using the previously described method, following exposure to clemastine fumarate at sublethal concentrations for a defined period of time.^17^ Metabolomic and lipidomic data analyses were performed using the PiMP^17^ pipeline for data filtering and metabolite annotation, as previously described.^17^

### Thermal Proteome Profiling (TPP)

For TPP analysis, *L. mexicana* was prepared following our previously described methods.^64^ In brief, cultures of *L. mexicana* parasites were prepared and underwent centrifugation steps to obtain a pellet, which was then washed with PBS 1× (pH 7.4, Gibco, Life Technologies) and resuspended in lysis buffer (50 mM monobasic potassium phosphate, 50 mM dibasic potassium phosphate, 0.5 M EDTA, 1 M DTT, 10 mM tosyl-l-lysyl-chloromethane hydrochloride, 0.8% *n*-octyl-β-d-glucoside, and mini protease inhibitor cocktail (EDTA-free)). Following freeze–thaw cycles and centrifugation, the lysate was obtained. Drug-induced disruption and heat treatment were performed on the lysate. Each lysate was divided into subsamples: 100 μM clemastine fumarate and a control (vehicle). For each condition, 250 μg of lysate was added to seven microcentrifuge tubes, with each tube representing a different temperature (37, 45, 50, 55, 60, 65, and 70 °C). The tubes were incubated for 3 min, followed by recovery of the soluble protein fraction through centrifugation. Alkylation and digestion of the proteins were carried out, and test samples and internal standard (*L. mexicana* MNYC/BZ/62/M379 strain) were labeled using a light and heavy dimethyl strategy for HPLC-MS/MS analysis, respectively. Data analysis was conducted using Thermo Proteome Discoverer and SEQUEST, with protein abundance normalized and melting curves analyzed to determine melting temperatures (*T*_m_) using GraphPad Prism 10. Heat maps (Figure S6) were generated to visualize the results (www.heatmapper.ca/expression) using its protein expression plugin with average linkage as the clustering method applied to rows and Euclidean as a distance measurement method. Hits with calculated *T*_m50_ outside the applied temperature range (37–70 °C) were manually removed (Table S5).

### Generation and Analyses of Transgenic Cell Lines

KO cell lines lacking the gene encoding *Lmx*IPCS (Gene ID: *LmxM.34.4990*; *Lmx*IPCS–/−) were generated using the CRISPR-Cas9 approach as described.^35^ All primers required for transgenic cell lines generation were designed using the primer design tool from www.LeishGEdit.net. For knockout cell line generation, the *Lmx*T7:Cas9 cell line was transfected with two repair DNA containing different drug resistance markers amplified from pTBlast and pTPuro plasmids and two sgRNA template products of the amplification using 5′ and 3′ gene-specific primers with the scaffold G00 primer. The products of the amplifications were purified by phenol:chloroform:isoamyl alcohol and precipitated using ethanol and the DNA pellet air-dried in a sterile environment before resuspension in 10 μL of sterile ultrapure water, quantification, and transfection. Briefly, *Lmx*T7:Cas9 cells was washed once with 1× PBS and adjusted to 8 × 10^6^ p/mL in Lonza P3 solution (Nucleofector Solution with supplement (4.5:1)). A mixture of 100 μL of cells and 20 μg of each repair DNA and sgRNA was transferred into the electroporation cuvettes, which was placed on ice for 10 min. Cuvettes were dried and electroporated using the FI-115 pulse code in the 4D Nucleofector system (Lonza). The transfected cells were recovered with 500 μL of FBS and transferred into a flask containing medium with 10% FBS. Drug pressure was applied 16 h post-transfection. KO clones were selected using a 96-well plate approach. *Lmx*IPCS was cloned into pRIB-mCherry-Neo using XhoI and NotI restriction enzymes (NEB) to create pRIB-*Lmx*IPCS. AB lines (*Lmx*IPCS–/–:*Lmx*IPCS) were generated by transfecting 100 μg of purified SspI enzyme (NEB) linearized pRIB-*Lmx*IPCS into cloned *Lmx*IPCS–/– as above. All transgenic lines were verified by PCR using Q5 High-Fidelity 2x Master Mix (NEB) and following the PCR program recommended by the manufacturer and Sanger sequence analyses. For RT-qPCR assays, RNA was extracted from *Lmx*T7:Cas9, *Lmx*IPCS–/–, and *Lmx*IPCS–/–:*Lmx*IPCS using Quick-RNA Miniprep Kit (Zymo Research). Subsequently, 0.5 ng of RNA was used in reactions containing 10 μM of each primer, 1 U of SuperScript III with Platinum (Invitrogen), 2× Reaction Mix containing SYBR Green in a 25 μL reaction volume. The PCR was run using the BioRad CFX Connect RT System.

### Lipid Analyses of Transgenic Cell Lines by Metabolic Labeling, Thin Layer Chromatography (TLC) and Liquid Chromatography–Mass Spectrometry (LC-MS)

The full dependence of IPC synthesis on IPCS was verified by metabolic labeling, lipid extraction,^65^ thin layer chromatography (TLC) and mass spectrometry, similarly to as previously described.^52^ In brief, cell pellets (10^8^) were washed with serum-free Schneider’s medium pH 7.0 and resuspended in 1 mL of the same in Protein-LoBind 1.5 mL tubes (Eppendorf), before incubation with 5 μM of C_6_-NBD-ceramide complexed to bovine serum albumen (AvantiPolar Lipids) for 3 h at 26 °C. After washing with TBS buffer the cells were resuspended in 750 μL ddH_2_O:CHCl_3_:MeOH (0.8:1:2) before incubation on ice with regular vortexing for 30 min. A biphasic separation was induced by adding 350 μL of CHCl_3_ and ddH_2_O, before centrifugation for 10 min at 3.000 rpm. The lower phases were transferred to other Protein-LoBind 1.5 mL tubes and dried under a vacuum before resuspending in 20 μL of CHCl_3_:MeOH (2:1). To separate the product, 10 μL was run-on high-performance TLC plates (Merck) and analyzed as previously described^66^ but using a Typhoon 9400 fluorescence scanner (λ_Ex_ 480 and λ_Em_ 540). Lipidomic analyses of cell extracts were performed by high resolution liquid chromatography–mass spectrometry (LC-MS) using an Exactive Orbitrap mass spectrometer (Thermo Scientific, Hemel Hempsted, UK) interfaced to a Thermo UltiMate 3000 RSLC system as previously described.^38^ Graphs were plotted with Prism v9.4 (GraphPad, San Diego, USA).

### Growth, Morphological Assessment, and Drug Sensitivity of the Cell Lines

To assess the growth and morphology of all cell lines of interest, procyclic promastigotes were monitored by counting in a Neubauer Hemocytometer (Merck) every 24 h for over 8 days. One ×10^5^ cells/mL were loaded into a 24-well plate, and every 3 days these were subcultured to maintain the parasites in log-phase. The phenotype of the transgenic cell lines was observed after fixing parasites with 4% of 16% formaldehyde solution methanol free (w/v) (Thermo Fisher). Dose–response experiments of the known anti-leishmanials clemastine fumarate, miltefosine, and amphotericin B (Sigma-Aldrich) were performed against the cell lines in 96-well plates. Initial concentrations of 25 μM clemastine fumarate, 100 μM miltefosine, and 10 μM amphotericin B were serially diluted in Schneider’s insect culture medium pH 7.0 and 1 × 10^6^ of procyclic promastigotes added per well before incubation for 20 h at 26 °C. DMSO and amphotericin B or miltefosine were used as a negative and positive controls. Subsequently, 10% resazurin sodium salt (Sigma) in PBS was added for 4 h at 26 °C and the plates read using a Biotek Synergy HTX fluorescence microplate reader (λ_Ex_ 560 and λ_Em_ 590). Experiments were performed in triplicate in three independent experiments.

### Functional Analyses Expressed, Isolated, and Proteosomal *Leishmania mexicana* and *L. major* Inositol Phosphorylceramce Synthase (*Lmx*IPCS and *Lmj*IPCS)

The expression of the *Lmx*IPCS and *Lmj*IPCS were performed using a cell-free system according to the manufacturer’s protocol (Cell-Free Sciences). Briefly, the gene encoding the IPCS was tagged with FLAG and cloned into pEU (GenScript) and then used in a transcription reaction at 37 °C for 6 h. The mRNA produced was then used for translation in the presence of artificial liposomes and wheat germ extract for 72 h at 15 °C. The resultant proteoliposomes were purified and protein expression confirmed by Western Blotting using a DYKDDDDK tag antibody (1:1.000; Thermo Fisher) and antimouse IgG (1:10.000) (BioRad). Enzyme activity was determined in the presence of the substrates phosphatidylinositol (PI) and the C_6_-NBD-ceramide (both AvantiPolar Lipids). Five μL of PI 1 mM was dried onto Protein-LoBind 1.5 mL tubes and 30 μL of proteoliposomes and 50 mM PO_4_ buffer pH 7.0 added before incubation for 10 min at 30 °C. The enzymatic reaction was then started by the addition of 5 μM of C_6_-NBD-ceramide in a 100 μL final reaction volume before incubation for 1 h at 30 °C. The reaction was quenched by the addition of 300 μL of 10:10:3 CHCl_3_:MeOH:H_2_O (HPLC grade) for 10 min at room temperature. Following drying under a vacuum, the samples were resuspended in 20 μL of CHCl_3_:MeOH:H_2_O and activity confirmed by fractionation on a high performance TLC plate and quantitation as above. For the evaluation of clemastine fumarate inhibition, the proteoliposomes and compound were preincubated for 1 h at 30 °C, followed by the protocol described above. The effect of the inhibitor on enzyme activity was determined using concentrations ranging from 0.03 mM to 2 mM.

### Quantitation of Inositol Phosphate Species

To quantify the total content of inositol phosphates (IPs) in *L. mexicana* procyclic promastigotes we adapted an LC-MS/MS-based method which enables baseline resolution for these analytes. *L. mexicana* procyclic parasites maintained in the exponential phase of growth were seeded (5 × 10^5^ parasites/mL) in 60 mL of Schneider’s insect medium pH 7.0 supplemented with 15% FBS and incubated at 26 °C for 72 h. Parasites were harvested by centrifugation (1,000*g* for 8 min) and washed twice with buffer A plus glucose (BAG; 116 mM NaCl, 5.4 mM KCl, 0.8 mM MgSO_4_, 50 mM HEPES-KOH, pH 7.2, and 5.5 mM d-glucose). The final pellet was resuspended in 1 mL of 1 M perchloric acid (PA) with 5 mM EDTA, mixed by vortex, and chilled on ice for 5 min. The cell homogenate was centrifuged (14,000*g* for 5 min at 4 °C) and the supernatant transferred to tube and kept on ice. Pellets were vacuum-dried and resuspended in 0.6 mL of buffer (8 M urea, 20 mM Tris-HCl pH 7.8 and 1 mM PMSF) for protein quantification using the bicinchoninic acid (BCA) protein assay kit (Thermo, Fisher). Supernatants were neutralized by addition of 194 μL of 1 M potassium carbonate added of 5 mM EDTA and then extracts treated with 275 nM of recombinant exopolyphosphatase (ScPPX1, UNIPROT entry: P38698; histidine tagged version expressed in *E. coli*(67) and purified by IMAC, before storage in 50 mM Tris-HCl, 150 mM NaCl, 10% glycerol, at pH 8.0). Following treatment for 30 min at 37 °C the reaction was stopped by the addition of 0.2 M PA solution. PPX1-treated extracts were then incubated with 5 mg of titanium dioxide (TiO_2_) beads for the enrichment of IPs and eluted with ammonium hydroxide (NH_4_OH) as previously described.^68^ The eluted IP fractions were concentrated (1 h at 60 °C) under a vacuum and 76 μL of MS-grade water plus acetonitrile added to each sample before storage at −20 °C until needed.

The IP content was profiled using an LC-MS/MS method to monitor inositol bisphosphate (IP_2_) and inositol hexakisphosphate (IP_6_). Samples were analyzed using a Nexera-X2 UHPLC (Shimadzu, Japan) connected to a 6500 QTrap (Sciex, Darmstadt, Germany). Instrument operation, data evaluation, and analysis were performed in Analyst version 1.7 (Sciex). Chromatographic separation was performed on a 5 μm HILICpak VG-50 column (Shodex, Tokyo, Japan) (2.0 × 150 mm), as described by Ito et al.,^69^ with a flow rate of 0.25 mL/min and an injection volume of 10 μL. Mobile phase A was 200 mM ammonium bicarbonate (NH_4_)HCO_3_ pH 10.0, adjusted with NH_4_OH, and mobile phase B comprised of 25% MeOH. The gradient was as follows: initial 0–1 min 100% B, to 75% B over 2 min, 65% B over 3 min, 55% B over 4 min, 25% B over 5 min and returned to 100% B over 3 min and held for 4 min. The ESI source was operated in negative electrospray ionization mode using multiple reaction monitoring (MRM). Source parameters were as follows: Curtain gas: 505, IonSpray voltage: −4500, TEM: 500°, GS1:40, and GS2:30. Two transitions were monitored per analyte, for IP_2_ quantifier transition was 338.9 > 241 and qualifier transition was 338.9 > 258.9, with a DP of −41.2v and CE of −31.3v. For IP_6_ the quantifier transition was 658.8 > 560.8 and qualifier transition 328.681 > 78.8, with a DP of −35v and CE of −36v and −84v, respectively. To avoid potential misidentification with soluble sugars we profiled glucose 1,6-bisphosphate (C_6_H_14_O_12_P_2_, *M*_r_ = 340.114 g mol^–1^) using this experimental procedure, and the verified retention time differs from IP_2_ (C_6_H_14_O_12_P_2_, *M*_r_ = 340.12 g mol^–1^).

### *In VitroLeishmania mexicana* Infection Assay

Mouse peritoneal macrophages (PEMs) were obtained from female CD-1 mice (8–10 weeks old) 1 day after starch induction (2% starch in sterile PBS, ip). They were collected through abdominal lavage with RPMI-1640 medium after which the PEMs were washed, counted, and resuspended in RPMI-1640 medium supplemented with 10% heat-inactivated fetal calf serum (FCS) to a density of 5 × 10^5^/mL. Aliquots of 100 μL of PEM suspension were transferred to each well of a 16-well Lab Tek slides, and the PEMs were left to adhere for 24 h at 37 °C in an atmosphere of 5% CO_2_ in air. The next day stationary phase *Leishmania* parasites (*Lmx*T7:Cas9, *Lmx*IPCS–/– and *Lmx*IPCS–/–:*Lmx*IPCS) were washed, resuspended in RPMI-1640 with 10% FCS, and added to the PEMs in a 1:1 ratio. The ability of the parasite lines to infect macrophages was evaluated microscopically after a period of 24 and 72 h. After each incubation period, the medium was removed from the respective slides followed by methanol fixation and Giemsa staining (10% Gurr solution in water). The percentage of infected macrophages was determined microscopically.

### Murine Infectivity of *Leishmania mexicana* Transgenic Lines

Following initial *in vivo* passage to ensure infectivity, each *L. mexicana* transgenic line was injected in the rump of BALB/c mice to evaluate their ability to infect mice and produce lesions. One day prior to infection, female BALB/c mice were shaven using electric clippers. The next day stationary phase *Leishmania* promastigotes were washed and resuspended to a density of approximately 2 × 10^8^/mL and 200 μL, then injected subcutaneously in the rump above the tail (*n* = 5 per *Leishmania* line). Mice were inspected twice weekly for the appearance of a small nodule on the rump. Once a nodule was visible it was measured with digital callipers in two perpendicular directions and the average diameter was reported. At the end of the experiment and after sacrifice of the mouse, the lesion nodule was removed and stored at −70 °C until further processing.

Skin parasite loads were quantified targeting a 170bp region in the *Leishmania* 18S ribosomal gene. The lesion nodule was halved, and the weight determined. The skin tissue was further cut in smaller pieces and transferred to a Precellys tube (CK28-R) together with 1 mL of sterile PBS. The tube was subsequently placed in a Precellys Evolution and homogenized in 3 cycles of 1 min at 6,500 rpm. The homogenate was briefly centrifuged at 100*g* to remove large tissue chunks and 50 μL of the supernatant was transferred for DNA extraction using the DNeasy blood & Tissue kit (Qiagen) according to the manufacturers guidelines and eluted in 50 μL of sterile Milli-Q water. The DNA of the samples and the standard curve were subsequently used in a qPCR reaction using primers and a FAM-probe targeting the 18S *Leishmania* kinetoplast DNA (FP: 5′-CCAAAGTGTGGAGATCGAAG-3′, RP: 5′-GGCCGGTAAAGGCCGAATAG – 3′ and probe: 6 FAM-ACCATTGTAGTCCACACTGC-NFQ-MGB). The standard curve was prepared by submitting 50 μL of the skin homogenate spiked with 1 × 10^8^*L. major* promastigotes to the same extraction procedure as the samples followed by 10-fold dilutions. The amplification reaction was performed in a 10 μL volume containing 1 μL of genomic DNA, 400 nM of each primer, 100 nM of probe and 5 μL of the 2× SensiFAST Probe mix (Bioline). PCR cycle conditions consisted of an initial denaturation step at 95 °C for 5 min, 40 cycles of 95 °C for 10 s and 60 °C for 40 s. The samples were analyzed in duplicate, and a standard curve, a no-template control, and a negative control were included in each run. The limit of quantification was established as 1000 *L. major* parasites per 50 μL skin homogenate. A one-way ANOVA with the Tukey post hoc test (*p* < 0.05) was performed to investigate statistical differences in skin parasite loads between the *Leishmania* lines.

## Supporting Information Available

Figure S1: In vitro evolution of drug resistance in *Leishmania mexicana* promastigotes during culture with clemastine fumarate in Schneiders media. Figure S2 Chromosomal Copy Number Variation (CNV) analyses of the two independent in vitro derived clemastine fumarate resistant clones (Lmx-cl-2 and Lmx-cl-4). Figure S3 SNPs identified in genes of the sphingolipid pathway in two CleR individual lines of L. mexicana promastigotes. Figure S4 (A) PCA plot showing the global differences between treated and untreated cells. Volcano plot showing significant and non-significant log2 fold-changes of metabolites peaks as detected by LC-MS (untargeted metabolomics) in mid-log wild type *Leishmania mexicana* promastigotes (1 × 10^8^) after treatment with clemastine fumarate (10 μM) for 12 h. Figure S5 (A) Relative abundance of clemastine sub-metabolites identified in biological replicates of treated (WTTx) and untreated parasites (WTCx). Figure S6 Heat map illustrating the thermal stability of soluble protein cell extracts from *Leishmania mexicana*. Figure S7 Relative abundance (*y*-axis) of TCA cycle metabolites identified in wild type *Leishmania major* promastigotes after clemastine fumarate (10 μM) exposure for 12 h. Figure S8 Enzymatic activity in *Leishmania mexicana* cell lines. Figure S9 LC-MS analyses demonstrated diminution of cardiolipins (CL) following add back. Table S1 Variants identified in two individual clones (LmxMcl.2 and LmxMcl.4) of clemastine resistant *Leishmania mexicana* promastigotes. Table S2 Polymorphisms found in genes involved in the sphingolipid pathway in two CleR-*Leishmania mexicana* clones. Table S3 Miltefosine and amphotericin B sensitivity of *Leishmania mexicana* parental (LmxT7:Cas9), LmxIPCS null (LmxIPCS–/−), and LmxIPCS addback (LmxIPCS–/–:LmxIPCS) lines. Table S5 Proteins detected in Leishmania mexicana subjected to clemastine fumarate treatment or untreated, revealing marked changes due to significant temperature variation >4°C (PDF)
Table S4 Excel file detailing identified mutations associated with clemastine fumarate pressure (XLSX)
Table S6 Excel file detailing LCMS data from the metabolomic analyses (XLSX)
