# Supplementary material for: Evaluation of the Leishmania Inositol Phosphorylceramide Synthase as a Drug Target Using a Chemical and Genetic Approach
Source: ACS Infect Dis. 2024 Jul 18;10(8):2913–28. doi: 10.1021/acsinfecdis.4c00284 (PMC11320567; doi:10.1021/acsinfecdis.4c00284)
Supplement: Supplementary file 1 — id4c00284_si_001.pdf [file id4c00284_si_001.pdf]

Evaluation of the *Leishmania* inositol phosphorylceramide synthase as a drug target using a chemical and genetic approach

Edubiel A. Alpizar-Sosa[1#], Flavia M. Zimbres[1#], Brian S. Mantilla[1], Emily A. Dickie[2] Wenbin Wei[1], Gabriela A. Burle-Caldas[1,3], Laura N. S. Filipe[1], Katrien Van Bocxlaer[4], Helen P. Price[5], Ana V. Ibarra-Meneses[6], Francis Beaudry[7], Christopher Fernandez-Prada[6], Philip D. Whitfield[2], Michael P. Barrett[2] and Paul W. Denny*[1]

[1]*Department of Biosciences, University of Durham, South Road, Durham, DH1 3LE, UK*

email p.w.denny@durham.ac.uk

[2]*School of Infection and Immunity, College of Medical, Veterinary and Life Sciences, University of Glasgow, Glasgow G12 8TA, UK*

[3]*Departamento de Bioquímica e Imunologia, Universidade Federal de Minas Gerais, Caixa Postal 486 - 31270-901, Belo Horizonte, Minas Gerais, Brazil*

[4]*York Biomedical Research Institute, Hull York Medical School, University of York, York YO10 5NG, UK*

[5]*School of Life Sciences, Keele University, Staffordshire, ST5 5BG, UK*

[6]*Département de Pathologie et Microbiologie, Faculté de Médecine Vétérinaire, Université de Montréal, Saint-Hyacinthe, Quebec J2S 2M2, Canada*

[7]*Département de Biomédecine, Faculté de Médecine Vétérinaire, Université de Montréal, Saint-Hyacinthe, Quebec J2S 2M2, Canada*

[#]*These authors contributed equally to this work*

**Corresponding author*

S1

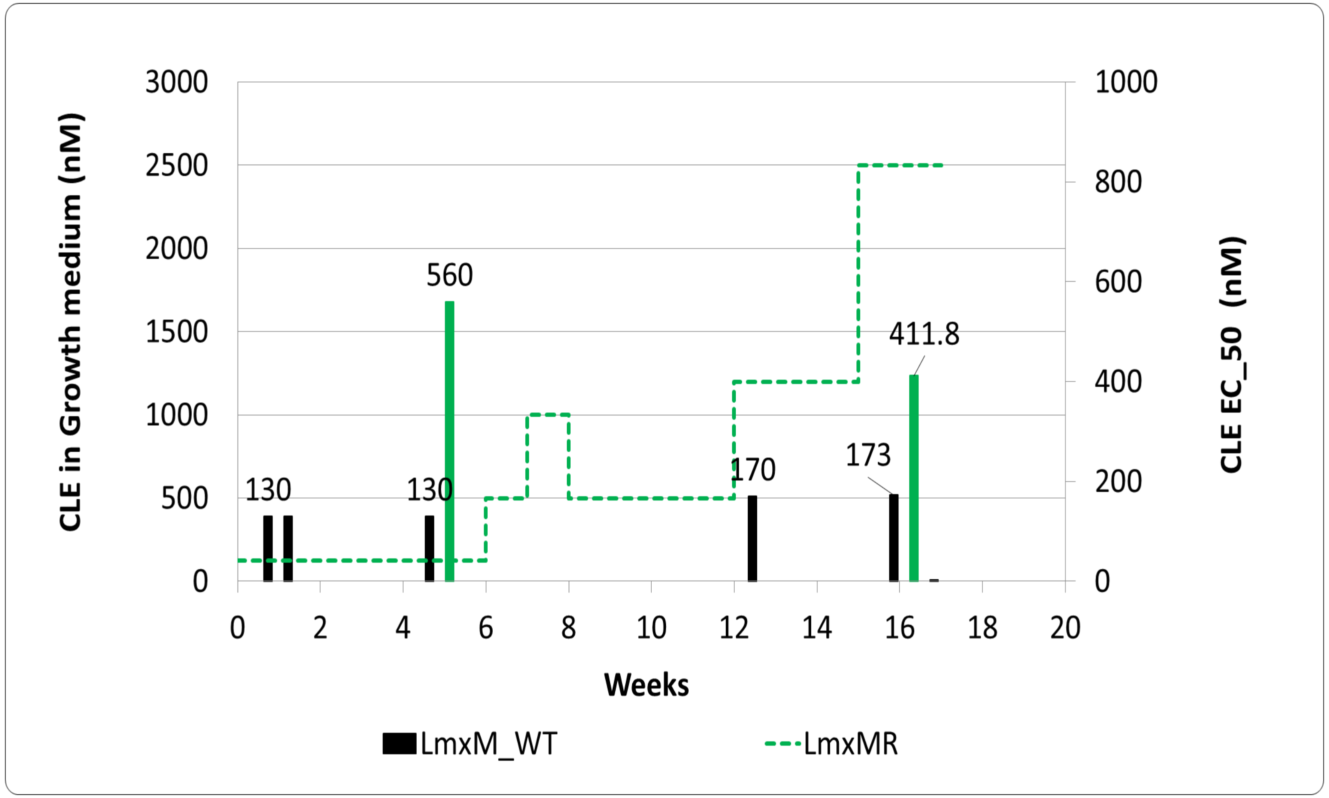

Figure S1 *In vitro* evolution of drug resistance in *Leishmania mexicana* promastigotes during culture with clemastine fumarate in Schneiders media. Figure shows values for wild type and one resistant clone (LmxM.cl.4). Lefthand *y-axis* shows concentration of clemastine fumarate (CLE) used in selection; righthand *y-axis* shows EC_{50} values at each point. Time of treatment in weeks – *x-axis*

S2

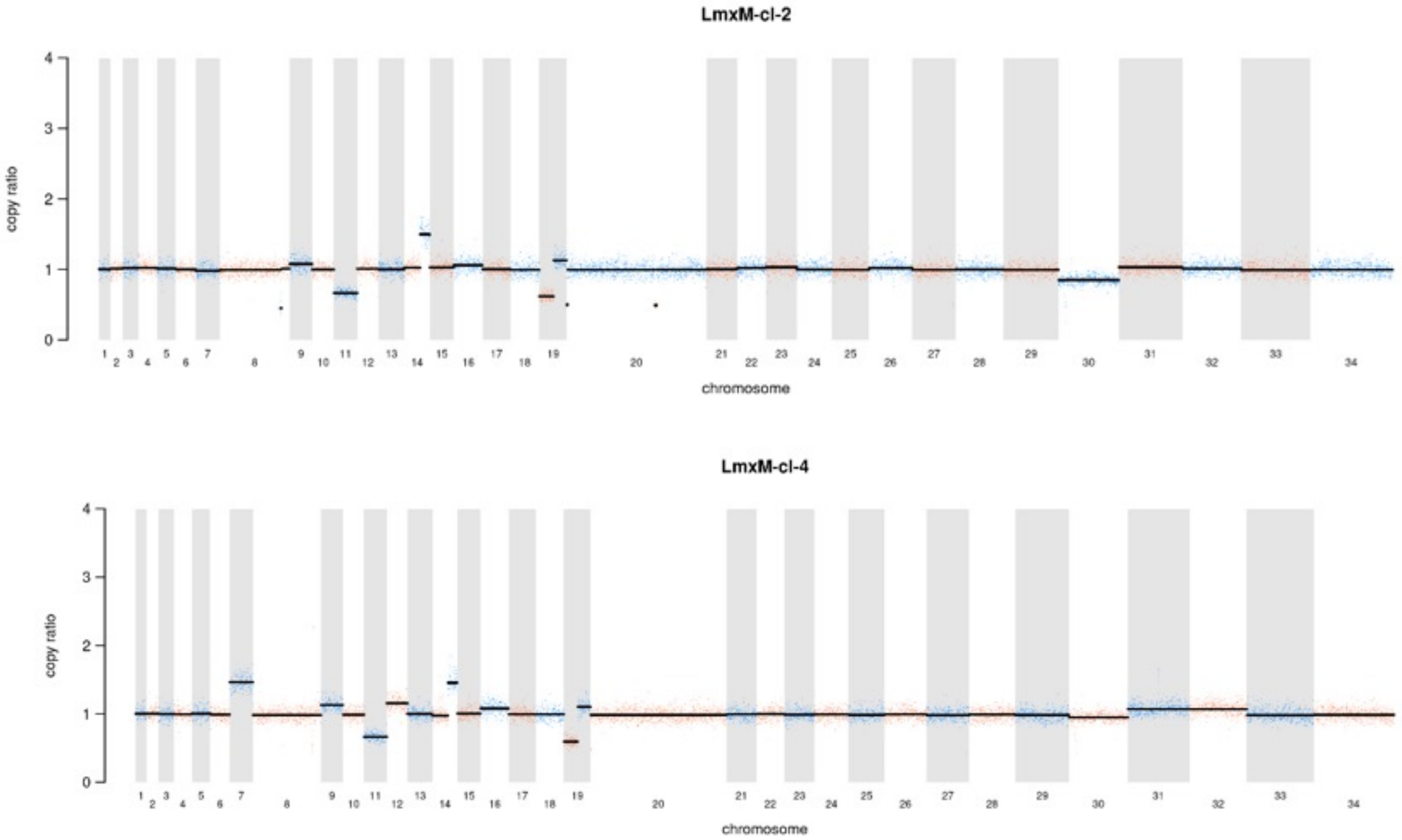

Figure S2 Chromosomal Copy Number Variation (CNV) analyses of the two independent *in vitro* derived clemastine fumarate resistant clones (Lmx-cl-2 and Lmx-cl-4). Copy number ratio *y-axis*; *Leishmania mexicana* chromosome number *x-axis*

S3

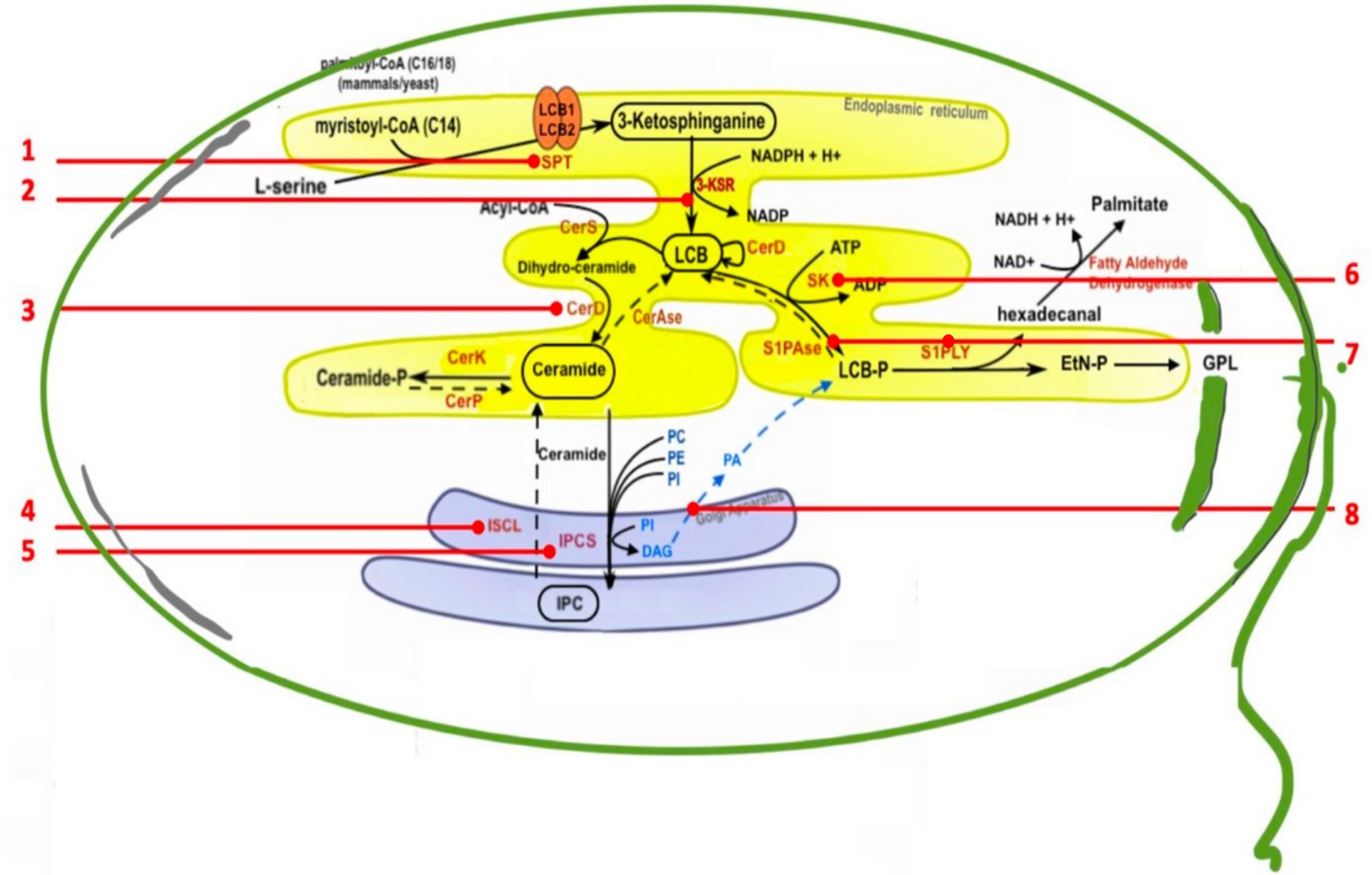

Figure S3 SNPs identified in genes of the sphingolipid pathway in two CleR individual lines of *L. mexicana* promastigotes. Numbers (red) highlight the genes in which SNPs were found. SPT (1); 3-KSR (2); CerD (3); ISCL (4); IPCS (5); SK (6); S1PAse and S1PLY (7); PAF (8). PC: phosphatidylcholine; PE: phosphatidylethanolamine; PI: phosphatidylinositol; DAG: diacylglycerol. A complete list of the enzyme names and SNPs in each gene is provided in Table S2 and Table S4 respectively.

S4

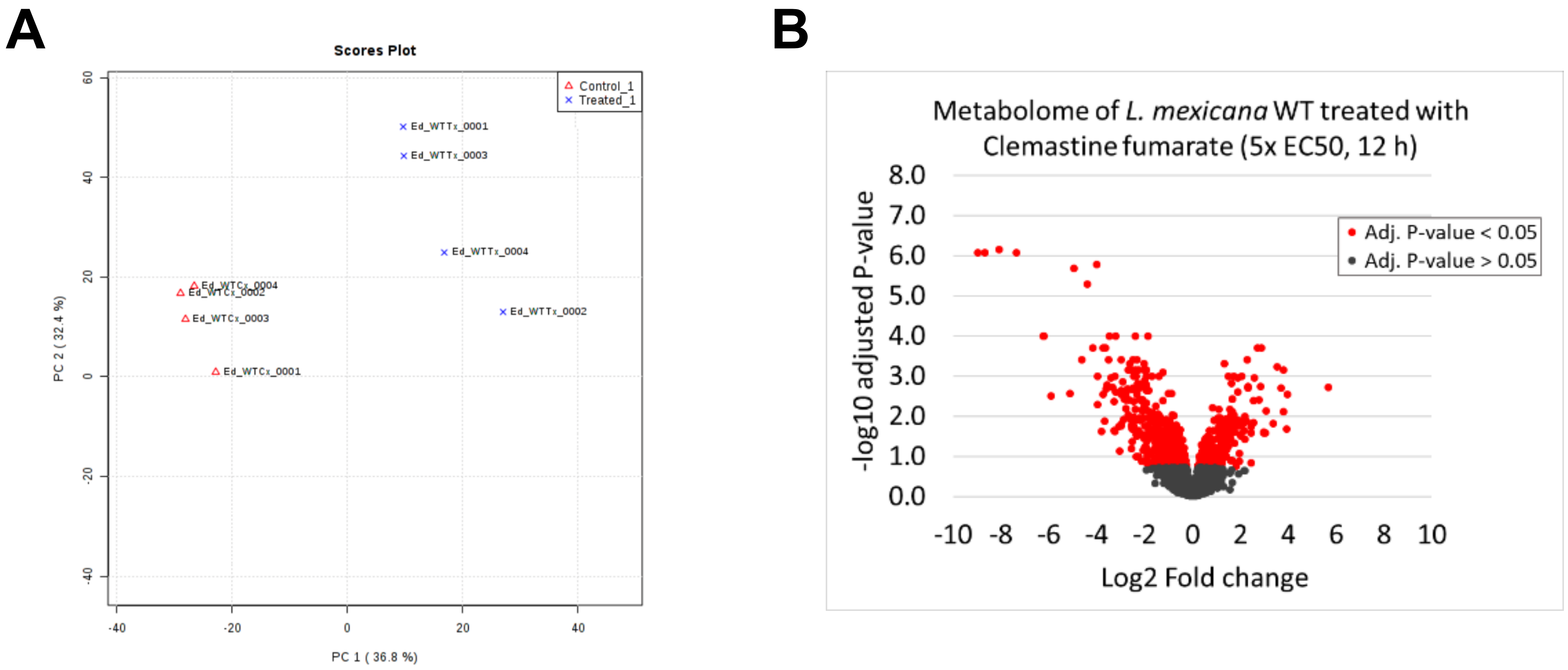

Figure S4 *(*A) PCA plot showing the global differences between treated (Treated_1, blue crosses) and untreated (Control_1, red triangles) cells. Dots represent biological replicates (n=4) of the measured metabolites. Clusters from both groups indicate good reproducibility across the machine run. Principal Components 1 and 2 are shown in *x*- and *y-axis*, respectively. (B) Volcano plot showing significant (n=341, red dots) and non-significant (n= 2,113, black dots) log2 fold-changes of metabolites peaks as detected by LC-MS (untargeted metabolomics) in mid-log wild type *Leishmania mexicana* promastigotes (1x 10^8) after treatment with clemastine fumarate (10 μM) for 12 h. Metabolomics and lipidomics analyses (LCMS) were performed and processed using multivariate data analysis with PiMP pipeline[58]. Note that each metabolite (peak) can also match several peaks, as several different structures with the same empirical formula may elute during the chromatographic separation (i.e. they will have different retention times) or may occur in multiple polarities (i.e. both positive and negative). For this reason, each data point may have more than one peak associated with it.

S5 **A**

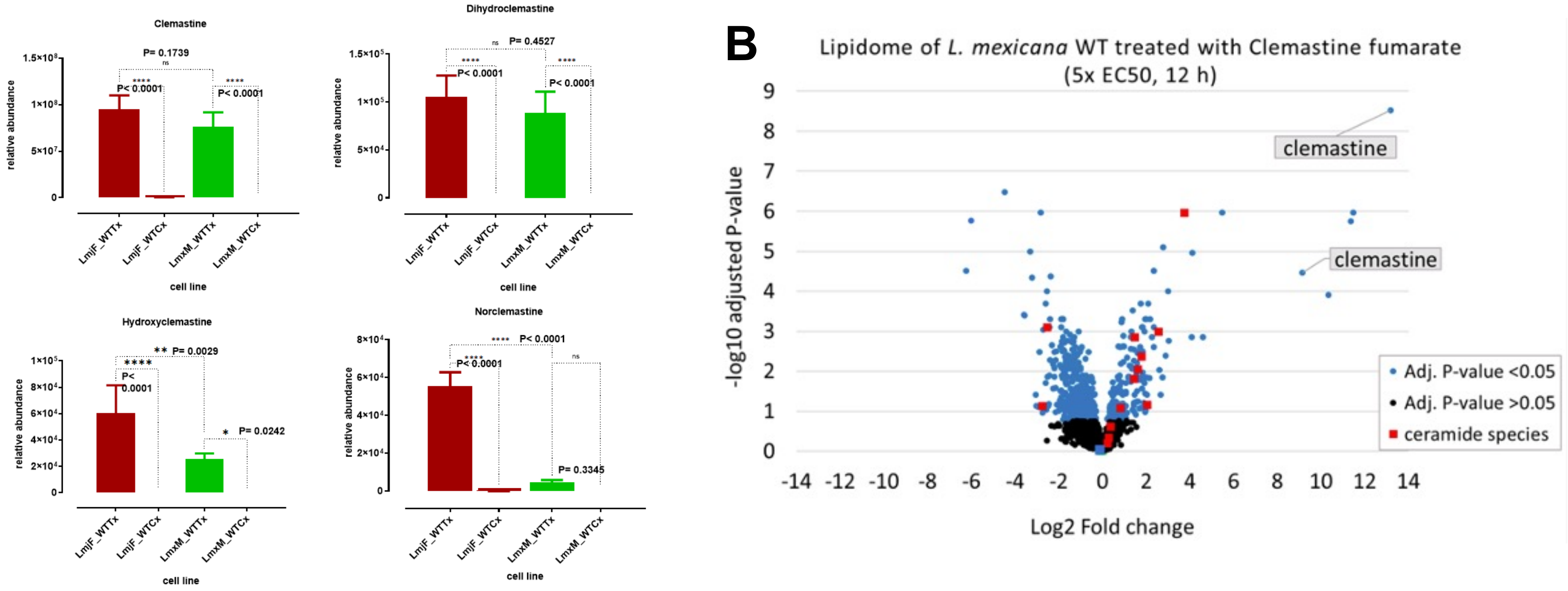

Figure S5 (A) Relative abundance (*y-axis*) of clemastine sub-metabolites identified in biological replicates (n=4) of treated (WTTx) and untreated parasites (WTCx) (*x-axis*). Metabolomics and lipidomics analyses (LCMS) were performed and processed using multivariate data analysis with PiMP pipeline[58]. The Benjamini-Hochberg procedure adjusted raw *P*-values (*q*-values) < 0.05 for ANOVA. Differences among samples (WTTx vs WTCx) were evaluated using one-way ANOVA with the Tukey's multiple comparisons test (*P*-value <0.05) using Prism software version 9.3.0. Statistically significant values ($P < 0.05$, 95% confidence interval) are shown with stars: ns, nonsignificant; *$P \leq 0.05$; **$P \leq 0.01$; ***$P \leq 0.001$; ****$P \leq 0.0001$). See Materials and Methods for details. Changes in other isomers peaks and the global metabolome are provided in Table S6. Reference values of clemastine fumarate and metabolites are from Tevell et al. 2010[59]. (B) Volcano plot showing significant (n= 314, blue dots) and non-significant (n= 1,720, black dots) log2 fold-changes of putative peaks as detected by LC-MS (targeted, lipidomics) in promastigotes after exposure to clemastine fumarate. Ceramides species are shown (red dots).

S6

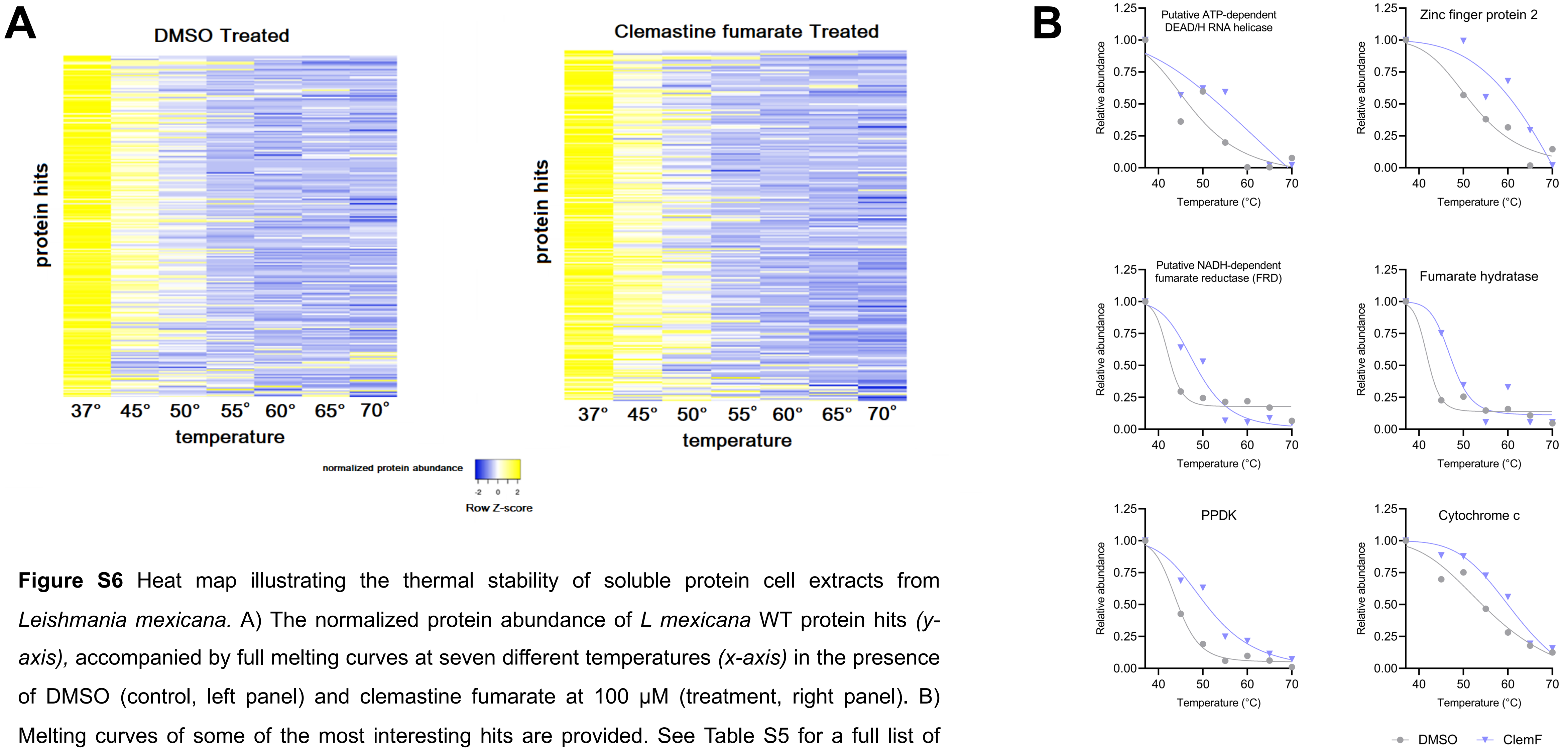

Figure S6 Heat map illustrating the thermal stability of soluble protein cell extracts from *Leishmania mexicana.* A) The normalized protein abundance of *L mexicana* WT protein hits *(y-axis),* accompanied by full melting curves at seven different temperatures *(x-axis)* in the presence of DMSO (control, left panel) and clemastine fumarate at 100 μM (treatment, right panel). B) Melting curves of some of the most interesting hits are provided. See Table S5 for a full list of protein hits detected in wild type *L. mexicana* using Thermal Proteomics Profiling (TPP).

S7

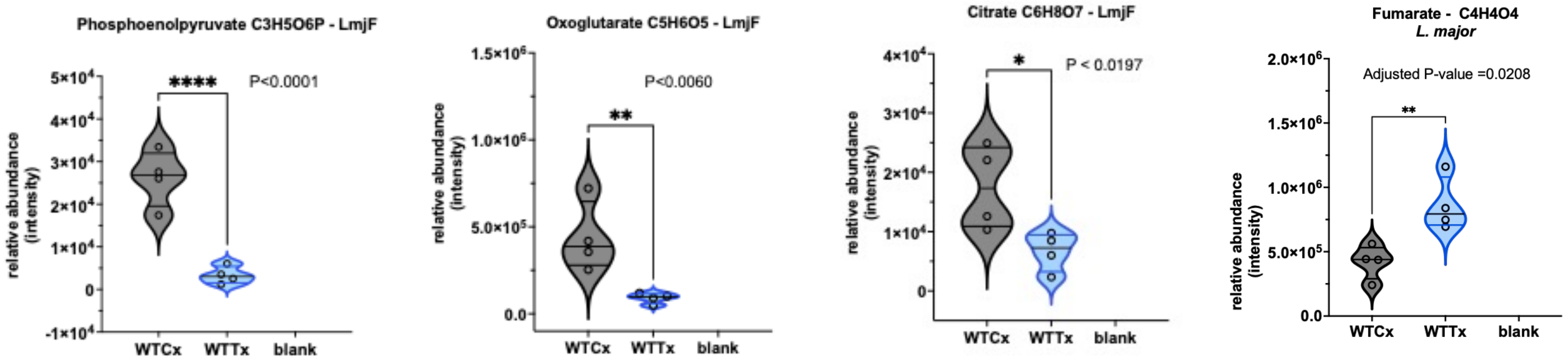

Figure S7 Relative abundance (*y-axis*) of TCA cycle metabolites identified in wild type *Leishmania major* promastigotes after clemastine fumarate (10 µM) exposure for 12 h. Biological replicates (n=4) of treated (WTTx) and untreated parasites (WTCx) (*x-axis*) were analysed with LCMS and processed using multivariate data analysis with PiMP pipeline[58]. The Benjamini-Hochberg procedure adjusted raw *P*-values (*q*-values) < 0.05 for ANOVA. Differences among samples (WTTx vs WTCx) were evaluated using one-way ANOVA with the Tukey's multiple comparisons test (*P*-value <0.05) using Prism software version 9.3.0. Statistically significant values (95% confidence interval) are shown with stars: ns, nonsignificant; $^{*}p \leq 0.05$; $^{**}p \leq 0.01$; $^{***}p \leq 0.001$; $^{****}p \leq 0.0001$). Changes in other metabolites are provided in Table S6.

S8

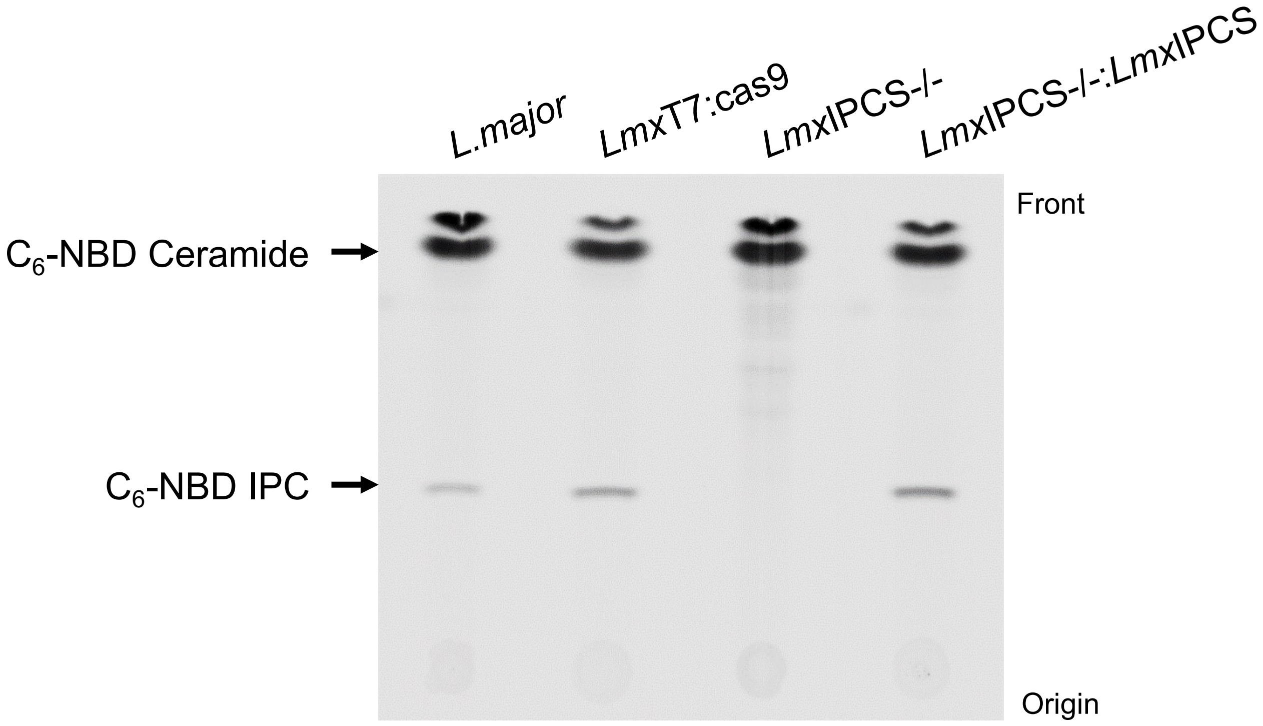

Figure S8 Enzymatic activity in *Leishmania mexicana* cell lines. Procyclic promastigotes were incubated with fluorescent ceramide (C_6-NBD Ceramide), followed by lipid extraction and analyses by TLC plate. The *Lmx*IPCS-/- mutant did not produce IPC, and this metabolic profile was restored upon reconstitution of the IPCS gene in the add-back line (*Lmx*IPCS-/-: *Lmx*IPCS). The *L. mexicana* parental line (*Lmx*T7:Cas9) and *L. major* FV1 were used as controls.

S9

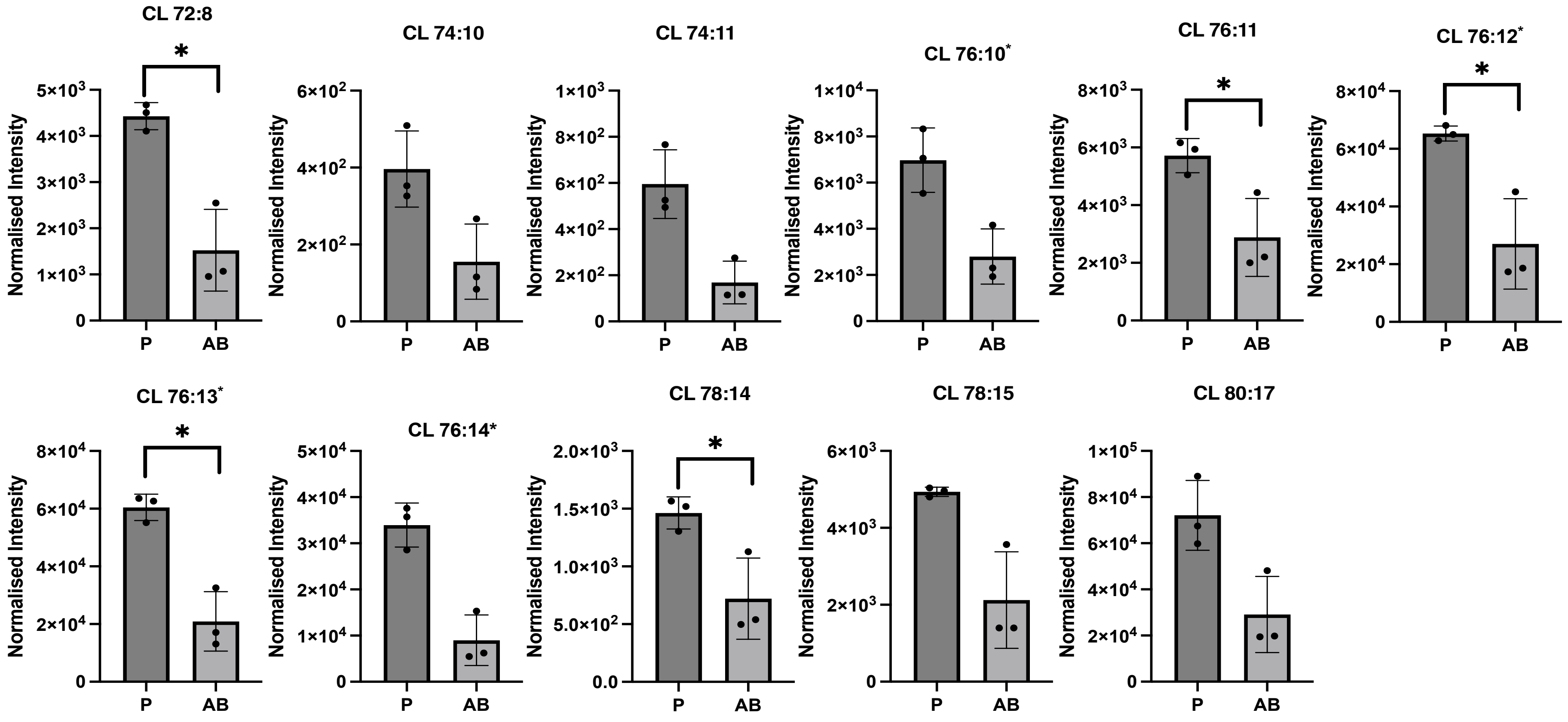

Figure S9 LC-MS analyses demonstrated diminution of cardiolipins (CL) following add back. Differences between samples parental (*Lmx*T7:Cas9; P) and add back (*Lmx*IPCS-/-:*Lmx*IPCS; AB) were evaluated using paired *t* tests in Prism software version 9.3.0. Statistically significant values are shown with stars: **p* ≤ 0.05. All data captured in the negation ion mode expect those with superscript asterisk which were captured in the positive ion mode.

S10

Table S1 Variants identified in two individual clones (LmxMcl.2 and LmxMcl.4) of clemastine resistant *Leishmania mexicana* promastigotes. IG: intergenic region (non-coding transcripts or splice region variants); CDS: coding sequence (missense or non-synonymous, silent (synonymous), start/stop lost/gained variants); AF: allele frequency.

**Clones**	**LmxMcl.2**	**AF**	**LmxMcl.4**	**AF**	**Total**
IG	13,152	0.02-1.0	12,892	0.02-1.0	26,044
(%)	44.24		43.36		87.61
CDS	1,932	0.02-0.67	1,750	0.02-0.73	3,682
(%)	6.49		5.88		12.38
Missense	1,582	0.02-0.67	1,425	0.02-0.73	3,007
(%)	5.32		4.79		10.11
Silent	349	0.03-0.67	322	0.02-0.53	671
(%)	1.17		1.08		2.25
TOTAL (n)	15,084	0.02-1.0	14,642	0.02-1.0	29,726
**(%)**	50.73		49.23		99.97

Table S2 Polymorphisms found in genes involved in the sphingolipid pathway in two CleR-Leishmania mexicana clones. Missense (non-synonymous) and silent SNPs in coding regions (CDS) are indicated with one (*) and two stars (**), respectively. Other SNPs correspond intergenic regions. Genes IDs are from the TritrypDB. SPT: serine palmitoyltransferase; 3-KSR: 3-dehydrosphinganine reductase; CerS: ceramide synthase; CerD: sphingolipid 4-desaturase; CerA: ceramidase; IPCS: inositol phosphorylceramide synthase; ISCL: inositol phosphosphingolipid phospholipase C; CerK: ceramide kinase; CerP: ceramide phosphatase; SK: sphingosine kinase; S1PAse: sphingosine-1-phosphate phosphatase; S1PLY: sphingosine 1-phosphate lyase; PAF: phosphatidic acid phosphatase. ND: non-determined. Numbered to map to Figure S3. Full details are shown in Figure S4.

	Gene ID	Alias	*L. mexicana* clone		Total of variants	Total of missense SNPs
			cl.2	cl.4		
1	LmxM.33.3740	SPT-like (LCB1)	1	2 / 1*	4	1
1	LmxM.34.0320	SPT (LCB2)		1**/ 1*	2	1
2	LmxM.34.0330	3-KDS	1	2	3	
	LmxM.30.1780	CerS (DHCS)				
3	LmxM.26.1670	CerD (SLDS)				
3	LmxM.26.1700	CerD (SLDS)		1	1	
3	LmxM.26.1690	CerD (SLDS)				
	ND	CerA				
4	LmxM.08.0200	ISCL	2	1 / 1*	3	1
5	LmxM.34.4990	IPCS	1	2	3	
	ND	CerK				
	ND	CerP				
6	LmxM.26.0710	SK	1	1	2	2
7	LmxM.31.2290	S1PAse	1**/ 1*	2*	4	3
7	LmxM.29.2350	S1PLY	1	2	3	
8	LmxM.18.0440	PAF	2	3	5	1
	**TOTAL**	**Total**	**11**	**19**	**30**	**9**

S12

Table S3 Miltefosine and amphotericin B sensitivity of *Leishmania mexicana* parental (LmxT7:Cas9), LmxIPCS null (LmxIPCS-/-) and LmxIPCS addback (LmxIPCS-/-:LmxIPCS) lines.

EC_{50} values shown in μM with 95% confidence intervals.

	**Miltefosine**	**Amphotericin B**
***Lmx*T7:cas9**	7.88 ± 0.34	0.31 ± 0.07
***Lmx*IPCS-/-**	15.24 ± 1.03	0.13 ± 0.03
***Lmx*IPCS-/-:*Lmx*IPCS**	7.17 ± 0.27	0.37 ± 0.09

S13

Table S4 Excel file detailing identified mutations associated with clemastine fumarate pressure

S14 **Table S5** Proteins detected in *Leishmania mexicana* subjected to clemastine fumarate treatment or untreated, revealing marked changes due to significant temperature variation ≥4°C.

Accession	Gene ID *L. mexicana*	Description	Tm_{50} (°C)		Δ*Tm* (°C)
			Clem	DMSO	
E9AU29	LmxM.36.5940	A distinct subfamily of CDD/CDA-like deaminases protein	64.32	37.96	26.36
E9AYZ0	LmxM.27.0880	Putative 2-oxoglutarate dehydrogenase subunit (OGDC) (or α-ketoglutarate dehydrogenase)	69.97	47.08	22.89
E9AZC6	LmxM.27.2200	Thioredoxin domain-containing protein	68.79	55.13	13.66
E9AL45	LmxM.07.0340	Putative ATP-dependent DEAD/H RNA helicase	59.21	46.69	12.52
E8NHP1	LmxM.15.0440a	tb-292 membrane associated protein-like protein	50.00	37.78	12.22
E9AYR5	LmxM.27.0130	Zinc finger protein 2	61.25	50.19	11.06
E9B5Z7	LmxM.34.1180	Putative NADH-dependent fumarate reductase (FRD)	48.3	38.38	9.92
E9B2E4	LmxM.31.0230	Dynein light chain, flagellar outer arm, putative	64.4	55.78	8.62
E9AKM1	LmxM.05.1140	V-type proton ATPase subunit	50.21	41.6	8.61
E9ALJ4	LmxM.08_29.2300	Ubiquitin carboxyl-terminal hydrolase	46.16	37.72	8.44
E9AU79	LmxM.36.6430	Protein transport protein SEC23	45.06	37.27	7.79
E9B686	LmxM.34.2080	Putative calcium motive P-type ATPase	51.75	44.2	7.55
E9B2L9	LmxM.31.0950	Staphylococcal nuclease homologue/Tudor domain containing protein, putative	45.37	37.99	7.38
E9AV49	LmxM.21.0810	methionine--tRNA ligase	44.68	37.35	7.33
E9B1Q4	LmxM.30.1070	Biotin/lipoate protein ligase-like protein	44.45	37.79	6.66
E9B2L2	LmxM.31.0880	60S ribosomal protein L18a	44.96	38.39	6.57
E9AVS1	LmxM.22.0890	Uncharacterized protein	44.51	37.97	6.54
E9B384	LmxM.31.3010	AMPK1_CBM domain-containing protein	49.7	43.3	6.40
E9AKF1	LmxM.05.0450	SKP1 component POZ domain-containing protein	44.39	38.01	6.38
E9ALK7	LmxM.08_29.2160	Rab GDP dissociation inhibitor	50.01	43.65	6.36
E9ATB8	LmxM.36.3390	60S ribosomal protein L29	44.83	38.48	6.35
E9ANG7	LmxM.11.0210	Inorganic diphosphatase	44.76	38.43	6.33
E9AV01	LmxM.21.0340	Mitochondrial processing peptidase alpha subunit,putative	44.71	38.42	6.29
E9ANC1	LmxM.10.1110	LsmAD domain-containing protein	54.74	48.53	6.21
E9AWM1	LmxM.24.0320	fumarate hydratase (FH)	47.76	41.72	6.04
E9B396	LmxM.31.3130	Putative ribosomal protein L3	49.72	43.71	6.01
E9AKM7	LmxM.05.1210	Protein phosphatase type 1 regulator-like protein	48.66	42.65	6.01
E9AZ73	LmxM.27.1710	Putative eukaryotic translation release factor	55.44	49.53	5.91
E9AYL4	LmxM.26.2330	Putative 60S ribosomal protein L35	50.62	45,00	5.62
E9ANP4	LmxM.11.1000	Pyruvate, phosphate dikinase (PPDK)	49.94	44.37	5.57
E9B5X5	LmxM.34.0950	PSP1 C-terminal domain-containing protein	42.88	37.35	5.53
E9ALB7	LmxM.07.1000	RNA binding protein-like protein	61.68	56.51	5.17
E9AQU2	LmxM.16.1310	Putative cytochrome c	59.16	54.2	4.96
E9AKT6	LmxM.06.0570	Putative 60S ribosomal protein L23a	48.64	44.28	4.36
E9AKP0	LmxM.06.0120	Peptidyl-prolyl cis-trans isomerase	52.11	47.76	4.35
E9ASX8	LmxM.36.2030	Chaperonin HSP60, mitochondrial	55.99	51.68	4.31
E9AW54	LmxM.23.0510	Uncharacterized protein	54.42	50.18	4.24
E9AVW5	LmxM.22.1330	Uncharacterized protein	50.07	45.95	4.12
E9AK27	LmxM.04.0460	Adenylosuccinate lyase	42.27	38.19	4.08
E9AKR5	LmxM.06.0370	Putative glutamine synthetase	41.58	37.5	4.08
E9B1G2	LmxM.30.0140	Ubiquitin carboxyl-terminal hydrolase	49.84	45.83	4.01

S15

Table S6 Excel file detailing LCMS data from the metabolomic analyses
